# Role of Oxygen Vacancies in Fe/Ru-Based Catalysts for the Reverse Water Gas Shift Reaction: Performance and Characterization

**DOI:** 10.1021/acsomega.5c11416

**Published:** 2026-01-27

**Authors:** Holly Dole, Gianni Caravaggio, Najmeh Ahledel, Ramzi Aoun, Hamid Radfarnia, Kourosh E. Zanganeh

**Affiliations:** Government of Canada, 6314 Natural Resources Canada, 1 Haanel Drive, Ottawa, Ontario K1A 1M1, Canada

## Abstract

The Reverse Water–Gas Shift (RWGS) reaction is a key process for converting carbon dioxide (CO_2_) into carbon monoxide (CO), enabling downstream synthesis of fuels and chemicals while contributing to CO_2_ emissions mitigation. This study investigates the performance of several Fe_*x*_-Ru_*y*_-based catalysts supported on different doped oxide materials (La–Al_2_O_3_, Ce–Al_2_O_3_, Sm-CeO_2_, Si–Al_2_O_3_), with the goal of identifying a cost-effective and thermally stable alternative to purely noble metal systems. Catalysts were evaluated in atmospheric conditions up to 800 °C and subjected to repeated temperature ramp cycles to assess CO_2_ conversion, CO selectivity, and long-term stability. Comprehensive characterization was performed using ICP-OES, BET, TPR, XRD, HRTEM, and XPS. The results reveal that optimizing the strength of the metal–support interaction, as well as the active metals ratio can have a significant impact, in terms of available active sites, which influences the catalytic performance, especially at lower temperatures (<500 °C). It was found that a ratio of 75% Fe and 25% Ru on Ce-doped Al_2_O_3_ provided this balance of properties. These findings provide insight into the design of robust, economically viable RWGS catalysts for efficient CO_2_ utilization.

## Graphical abstract

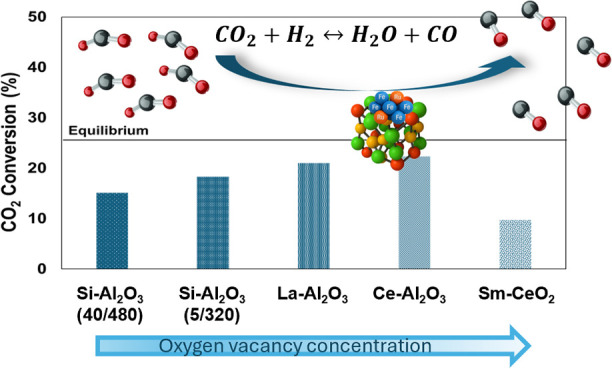

## 1 Introduction

With average atmospheric carbon dioxide (CO_2_) concentrations reaching 419 ppm in 2023,^1^ the mitigation of CO_2_ emissions has become a critical priority in global climate change efforts. It has been largely accepted that to mitigate climate change, carbon neutrality must be achieved, either by reducing reliance on fossil fuels through alternative energy source or treating the resulting emissions. One approach to treat emissions is through the combination of post-combustion carbon capture and catalytic CO_2_ conversion to useful intermediates or value-added products such as syngas, methanol, diesel, or gasoline.^2^ Compared to Carbon Capture & Storage (CCS), Carbon Capture & Utilization (CCU) does not treat CO_2_ as a waste, but rather as a feedstock and closes the anthropogenic carbon cycle, creating what is referred to as the “circular carbon economy”. CCU is both economically attractive and adaptable with the infrastructure that is currently in place.^2^

The reverse water–gas shift (RWGS) reaction, when combined with the Fischer–Tropsch (F-T) reaction, has become one of the more widely studied CO_2_ conversion pathways to produce fuels.^2–5^ When reacted with renewable hydrogen, CO_2_ can be converted into carbon monoxide and water via an equilibrium reaction.^3^ However, this process faces key challenges; it is highly energy intensive (1) due to its endothermic nature, and it is less thermodynamically favorable than the Sabatier reaction (2) at lower temperatures. Heterogeneous catalysts for CO_2_ conversion to fuels and chemicals, have been shown to improve reaction efficiency and performance.^4–14^ Separation (ex. pressure-swing adsorption, PSA) of CO_2_ and CO using an adsorbent can further improve process efficiency by recycling unreacted CO_2_.^15^ The RWGS reaction is typically carried out at temperatures up to 800 °C and atmospheric pressure. Other side reactions that can occur include CO methanation (3), methanol synthesis (4) and carbon deposition, via the Boudouard reaction (5), which can result in the deactivation of the active sites on the surface of the catalyst.^16^ Therefore, catalyst stability is another challenge in successfully implementing the RWGS pathway.

1 $$CO_{2}+H_{2}↔CO+H_{2}O{\Delta H}_{r298\;K}^{0}=41.2\frac{kJ}{mol}$$

2 $$CO_{2}+4H_{2}↔CH_{4}+2H_{2}O{\Delta H}_{r298\;K}^{0}=-164.6\frac{kJ}{mol}$$

3 $$CO+3H_{2}↔CH_{4}+H_{2}O{\Delta H}_{r298\;K}^{0}=-206.5\frac{kJ}{mol}$$

4 $$CO_{2}+3H_{2}\to CH_{3}OH+H_{2}O{\Delta H}_{r298\;K}^{0}=131.0\frac{kJ}{mol}$$

5 $$2CO↔CO_{2}+C{\Delta H}_{r298\;K}^{0}=172.0\frac{kJ}{mol}$$

The RWGS reaction has been studied across various thermo-catalytic systems. Triviño et al.^16^ provide a comprehensive overview of these studies and recent technological advances in order to improve the catalytic performance for the RWGS reaction as a practical way to utilize CO_2_. While most efforts target high CO yield and selectivity, such outcomes are generally attained only at elevated temperatures (>500 °C). A previous RWGS bench-scale study has shown CO_2_ conversions up to 69%, however, the CO selectivity only reaches 30%.^8^ On the other hand, investigations that have shown 100% CO selectivity report slightly lower CO_2_ conversions (approximately 20–40%).^10,14^ Many of the catalysts considered are metals or metal oxides supported on an O_2_-conductive material (e.g., yttria-stabilized zirconia (YSZ), ceria (CeO_2_), samarium-doped ceria (SDC)).^6,8,13,14,17,18^ Oxygen vacancies in these support materials are linked to higher CO_2_ conversion and selectivity. They enhance the adsorption of both CO_2_ and H_2_, thereby facilitating CO_2_ reduction on the catalyst surface. Additionally, these vacancies weaken the bond between the produced CO and the surface, which makes it easier for the CO to be released.^19–22^ At the same time, the presence of oxygen vacancies often give rise to strong metal–support interactions (SMSI). This property is important to consider when developing a catalyst for RWGS as stronger interaction does not always result in higher catalytic activity, due to the possibility of encapsulating the active metal(s).^23^

The influence of support materials on CO_2_ conversion with oxygen vacancies, as well as the influence of metal–support interaction, was shown in a previous study.^13^ The same work also examined the effect of mono- vs bimetallic nanocatalysts (Ru, Ru_20_Fe_80_, Ru_45_Fe_55_, Ru_80_Fe_20_), metal loading (0.5–5 wt %), and feed ratio (0.5:1 to 5:1). According to the study, the best performing catalyst was 2 wt % Ru_45_Fe_55_/Sm-CeO_2_, a bimetallic nanocatalyst, showing CO_2_ conversion close to equilibrium. The need for further investigation into the role of oxygen vacancies was acknowledged. The benefits of bimetallic nanocatalysts are noted as not only increasing catalytic performance through a synergistic effect, but also improving selectivity, by enhancing the adsorption of CO_2_, and stability, by harnessing the benefits of the precious metal properties at high temperatures.^24^

The purpose of our research is to provide a more in-depth comparison of the role of supported mono- and bimetallic nanoparticle catalysts for the RWGS reaction. In particular, the influence of support material and oxygen vacancies on CO_2_ conversion, CO selectivity and catalyst stability were examined, with the objective of achieving enhanced performance at lower temperatures. Three different structural variables were investigated: (i) the deposition order of Ru and Fe on a La-doped Al_2_O_3_ support, (ii) the Fe:Ru mass ratio on the same support, and (iii) the effect of support material and oxygen vacancies. In addition, the influence of different H_2_:CO_2_ feed ratios was examined.

While Fe–Ru bimetallic catalysts and Ce-modified alumina supports have been widely studied for CO_2_ conversion, most prior work has focused on methanation or single-support systems for RWGS. For example, Panaritis et al.^13^ examined RuFe nanoparticles on ceria-based supports for RWGS, and Iqbal et al.^25^ along with Price^26^ reported Ru–Fe–Ce/Al_2_O_3_ catalysts achieving near-complete methanation selectivity. In contrast, this study identifies a new performance window for Fe–Ru/Ce-Al_2_O_3_ catalysts under RWGS conditions, particularly at lower temperatures (e.g., 500 °C), where high CO selectivity is achieved at significantly lower Ru loadings. Furthermore, we provide insights into the role of oxygen vacancies in the support and their interaction with metal active sites, as well as the synergistic properties of the Fe–Ru bimetallic system using detailed TPR analysis. A systematic evaluation of supports with varying oxygen vacancy concentrations, combined with detailed characterization (BET, TPR, XPS), offers a level of understanding not previously reported. These findings contribute to both fundamental knowledge and practical considerations for RWGS catalyst design and synthesis for scale-up applications.

## 2 Experimental Section

### 2.1 Synthesis of Supported Nanoparticle Catalysts

Thirteen different Fe_*x*_Ru_*y*_ (2 wt % - based on a previous study^13^) nanocatalysts (Table 1) supported on La-doped Al_2_O_3_ (La–Al_2_O_3_; 10% lanthanum oxide, 90% alumina; specific surface area (SSA) = 143 m^2^·g^–1^), Sm-doped CeO_2_ (Sm-CeO_2_; 20% samarium oxide, 80% ceria; SSA = 203.1 m^2^·g^–1^), Ce-doped Al_2_O_3_ (Ce–Al_2_O_3_; 20% ceria, 80% alumina; SSA = 156 m^2^·g^–1^), and two Si-doped alumina supports (Si–Al_2_O_3_ (40/480); 40% silica, 60% alumina; SSA = 433 m^2^·g^–1^; and Si–Al_2_O_3_ (5/320); 5% silica, 95% alumina; SSA = 322 m^2^·g^–1^), were synthesized using incipient wetness impregnation (IWI). Three of the bimetallic catalysts were synthesized to evaluate the best approach for depositing the metal on the La-doped alumina support, that is, both Ru and Fe were deposited simultaneously (Ru_45_Fe_55_(SIM)/La–Al_2_O_3_), Ru was deposited first, then Fe (Fe_55_–Ru_45_), and Fe was deposited first, then Ru (Ru_45_–Fe_55_/La–Al_2_O_3_), respectively. These approaches were explored to see if the deposition order affected the available active sites and interaction between the metal nanoparticles.

**Table 1:** Metal Loading and Surface Area of 2 wt % Supported Fe_*x*_–Ru_*y*_ Catalysts

	**metal loading** **(wt %)** ^a^			**surface area** **(m^2^ g^–1^)** ^b^	
**catalyst**	**Fe**	**Ru**	**total**	**before**	**after**
Ru/La–Al_2_O_3_		1.99 (2)	1.99 (2)	141.7	
Fe_25_–Ru_75_/La–Al_2_O_3_	0.62 (0.51)	1.38 (1.54)	2.00 (2.05)	134.5	
Ru_45_–Fe_55_/La–Al_2_O_3_	1.52 (1.10)	0.74 (0.89)	2.26 (1.99)	137.6	
Ru_45_Fe_55_(SIM)/La–Al_2_O_3_	1.47 (1.10)	0.57 (0.92)	2.04 (2.02)	146.6	
Fe_55_–Ru_45_/La–Al_2_O_3_	1.55 (1.11)	1.01 (0.90)	2.56 (2.01)	130.8	
Fe_75_–Ru_25_/La–Al_2_O_3_	1.48 (1.49)	0.63 (0.56)	2.11 (2.05)	135.2	123.5
Fe_80_-Ru_20_/La–Al_2_O_3_	2.07 (1.62)	0.31 (0.20)	2.38 (1.82)	133.9	
Fe_90_-Ru_10_/La–Al_2_O_3_	1.98 (1.83)	0.13 (0.10)	2.11 (1.93)	134.1	
Fe/La–Al_2_O_3_	2.20 (2.02)		2.20 (2.02)	136.8	
Fe_75_–Ru_25_/Sm-CeO_2_	1.86 (1.53)	0.33 (0.51)	2.19 (2.04)	86.1	8.4
Fe_75_–Ru_25_/Ce–Al_2_O_3_	1.72 (1.52)	0.57 (0.49)	2.29 (2.01)	161.4	161.9
Fe_75_–Ru_25_/Si–Al_2_O_3_ (40/480)	1.73 (1.63)	0.66 (0.51)	2.39 (2.14)	342.8	
Fe_75_–Ru_25_/Si–Al_2_O_3_ (5/320)	1.91 (1.50)	0.68 (0.53)	2.59 (2.03)	278.2	

a Measured by SEM-EDX (nominal loadings in brackets).

b Measured by BET.

To evaluate the effect of Fe:Ru ratio, a range of monometallic (Ru or Fe only) and bimetallic catalysts were synthesized by adjusting the amount of metal precursor added. Finally, the effect of the support material was assessed by depositing the same ratio of Fe:Ru (75:25) on five different materials – with varying levels of oxygen vacancies. Ruthenium chloride trihydrate (RuCl_3_·3H_2_O; ≥99.9% trace metals basis) and iron­(III) nitrate nanohydrate (Fe­(NO_3_)_3_·9H_2_O; 98.0 to 101.0%) were used to prepare the catalysts by dissolving precursor salts separately in deionized water and depositing on the support consecutively (Fe_*x*_–Ru_*y*_ or Ru_*x*_–Fe_*y*_) or by dissolving both the precursor salts (in the case for Fe_45_Ru_55_(SIM)) in deionized water before depositing on the support, followed by drying at 120 °C for 8 h, and calcination at 550 °C for 6 h. It should also be noted that a total metal loading of 2 wt % was chosen based on a previous study that showed this loading to be an optimal amount for good catalytic performance.^13^

### 2.2 Catalyst Characterization

To determine the specific surface area of the samples the Brunauer–Emmett–Teller (BET) method was carried out using a Micromeritics ASAP 2020 surface area and porosity analyzer. Each sample was degassed at 250 °C for 16 h. Subsequently, the isotherms were measured by standard nitrogen adsorption at −196 °C.

Catalyst compositions were determined using a Hitachi S3400N VPSEM with an Oxford INCA energy-dispersive X-ray (EDX) detector system operating at 20 kV and 80–90 mA. A thin layer of powder catalyst was deposited and held in place on a double-sided carbon tape. The excess was blown off with a gentle stream of nitrogen. For each catalyst, ten random locations, chosen by the INCA automation software, were analyzed. Average and standard deviation of the measurements were calculated to determine the final composition of the catalysts.

X-ray powder diffraction (XRD) patterns were collected using a Rigaku Ultima IV XRD automated spectrometer over the angular range of 3–90° 2θ, with a step size of 0.05° and a scanning rate of 1°·min^–1^. The system operates in the θ:θ geometry, utilizing Cu Kα radiation (λ = 1.5405981 Å) and equipped with a diffracted-beam monochromator and a high-speed semiconductor one-dimensional X-ray detector (D/teX Ultra). Diffraction peaks of crystalline phases were identified using the International Center for Diffraction Data (ICDD) database and Jade Plus Version 7.5 software.

Temperature-programmed reduction by hydrogen (H_2_-TPR) was performed to evaluate the reducibility characteristics of the catalyst and how the bimetallic interaction affected this property. This was done using a Micromeritics AutoChem II 2920 Analyzer equipped with a thermal conductivity detector. Each catalyst (50 ± 2 mg) was pretreated in He at 30 mL min^–1^ and 400 °C for 30 min. The sample was then cooled to room temperature in the same He flow. The H_2_-TPR measurement was carried out by heating to 800 °C at a heating rate of 10 °C·min^–1^ in a 50 mL·min^–1^ flow of 10 vol % H_2_ and a balance argon gas mixture.

Selected catalysts were characterized by transmission electron microscopy (TEM) to directly image the catalyst on the supports both before and after catalytic testing. In both cases, the specimens were prepared by dispersing the solid powder in ethanol, and sonicating for 5 min. One drop of the solution was then placed onto a 200 mesh TEM copper grid coated with a lacey carbon support film (Ted Pella) and dried in air. The analysis was performed on a FEI Titan3 80–300 TEM operated at 300 keV and equipped with a CEOS aberration corrector for the probe forming lens and a monochromated field-emission gun was used to acquire high-resolution TEM (HRTEM). Average particle sizes for these catalysts were analyzed using ImageJ software. Due to the limited number of TEM images available, a statistically robust particle size distribution could not be obtained; therefore, only average values are reported.

The X-ray photoelectron spectroscopy (XPS) analysis of samples, as received, was performed using a Kratos Axis Ultra DLD spectrometer using a monochromatic Al K­(α) source (1486.7 eV) at 140 W. Survey and high-resolution scans were carried out with an analysis area of 300 × 700 μm and pass energies of 160 and 40 eV respectively. The Kratos charge neutralizer system was used on all specimens. The binding energy scale was calibrated using the main line of the carbon 1s spectrum set to 284.8 eV. Spectra were analyzed using CasaXPS Software (version 2.3.18).

The weight loss of the catalyst, before and after aging tests was measured from 25 to 800 °C in increments of 2 °C·min^–1^ using thermal gravimetric analysis (TGA). The purpose of using this technique was to gain insights into possible carbon deposition.

### 2.3 Experimental Setup and Operation

The catalysts were tested in a fixed-bed flow reactor system consisting of a vertical quartz tube (6.12 mm nominal ID by 40.64 cm long) enclosed in a temperature-controlled furnace as described previously.^27^ Approximately 200 mg of each catalyst were loaded into the quartz reactor tube. The catalytic performance was evaluated through temperature gradient experiments from 150 to 800 °C at 2 °C·min^–1^, under continuous flow conditions, for three temperature ramp cycles, at atmospheric pressure. This approach was selected based on preliminary validation tests to ensure that the measured conversions represent quasi-steady-state behavior rather than transient responses. Specifically, step-change experiments were performed by increasing the temperature in 50 °C increments and monitoring CO_2_ conversion until stabilization. These tests showed that steady-state was achieved within approximately 25 min under the studied conditions (feed composition, flow rate, catalyst mass). A representative stabilization profile is provided in the Supporting Information (Figure S1). Therefore, the temperature-gradient data reported herein can be considered quasi-steady-state, supported by these validation experiments.

It should be noted that the third ramp cycles of each catalyst are shown for the results, unless otherwise specified. All catalytic experiments were carried out with a w8 hly space velocity (WHSV) of approximately 135 h^–1^. The total flow was 400 mL·min^–1^, consisting of approximately 40 mL·min^–1^ CO_2_ (Grade 4.0), 40–120 mL·min^–1^ H_2_ (Grade 5.0) and the balance N_2_ (Grade 5.0) varying the ratio of H_2_:CO_2_ from 1:1 to 1:3. The stability of select catalysts were evaluated at a reactor temperature of 500 °C for 60 h using a 1:1 ratio of H_2_:CO_2_ after the third ramp cycle was completed.

From the influent and effluent gas compositions, the CO_2_ conversion ([Disp-formula eq5]) and CO selectivity ([Disp-formula eq6]) were calculated using the following equations, respectively:

6 $${\mathrm{CO}}_{2}\;\mathrm{conversion}\;\left(\%\right)=\frac{{\left[{\mathrm{CO}}_{2}\right]}_{\mathrm{in}}-{\left[{\mathrm{CO}}_{2}\right]}_{\mathrm{out}}}{{\left[{\mathrm{CO}}_{2}\right]}_{\mathrm{in}}}\times 100\%$$

7 $$CO\,\;selectivity\;\left(\%\right)=\frac{{\left[CO\right]}_{out}}{{{\left[{CO}_{2}\right]}_{in}-\left[{CO}_{2}\right]}_{out}}\times 100\%$$

## 3 Results and Discussion

### 3.1 Catalyst Characterization

The metal loading and BET surface areas of all the catalysts before testing and after testing for select catalysts are shown in Table 1. Nominal loadings, shown in brackets, were determined from the actual mass of precursor salt added with respect to the mass of support material during the synthesis.

The elemental composition of Fe and Ru on the catalyst support materials was analyzed using SEM-EDX. The measured metal loadings were generally consistent with the nominal values and target Fe:Ru ratio, indicating successful incorporation of the metals during synthesis. Notably, the Fe content was slightly higher than the nominal loading, which is attributed to surface enrichment of Fe. This observation is consistent with the synthesis method employed and the surface-sensitive nature of SEM-EDX.

When supported on La–Al_2_O_3_ (143 m^2^·g^–1^) and Ce–Al_2_O_3_ (156 m^2^·g^–1^), the presence of Fe/Ru did not change the surface area significantly (i.e., up to 13 m^2^·g^–1^). This is due to the high surface area and stable mesoporous structure of Al_2_O_3_, allowing to accommodate the loaded metal catalysts and indicates that the metal nanoparticles are well dispersed over the surface of the support. After aging tests, the Fe_75_–Ru_25_/Ce–Al_2_O_3_ catalyst did not show a significant change in surface area, while the Fe_75_–Ru_25_/La–Al_2_O_3_ showed a slight decrease (11.7 m^2^·g^–1^).

When supported on Sm-CeO_2_ (203.1 m^2^·g^–1^), the surface area of the loaded support decreased by over half (i.e., 117 m^2^·g^–1^). Furthermore, after testing, the surface dropped to 8.4 m^2^·g^–1^. This significant drop is a contributing factor that will be discussed further with the resulting catalytic performance; however, it is attributed to a decreased thermal stability on Sm-CeO_2_ and SMSI between Fe/Ru and Sm-CeO_2_, as confirmed from TPR and TEM results. The SMSI may have caused blocked or collapsed pores due the high oxygen mobility, resulting in such significant decrease in the surface area. Similarly, when supported on Si–Al_2_O_3_ (40/480) and Si–Al_2_O_3_ (5/320), the surface areas decreased to 433 m^2^·g^–1^ and 322 m^2^·g^–1^, corresponding to reductions of 90 and 44 m^2^·g^–1^, respectively. This decrease also contributed to their lower CO_2_ conversion, as discussed later.

To further investigate their structural properties, a full scan of the fresh catalysts was carried out using XRD (Figure 1). For the La–Al_2_O_3_ supported catalysts (Figure 1a), alumina (Al_2.667_O_4_) was identified as the predominant crystalline phase, with characteristic peaks at 19.5, 32.0, 37.2, 39.6, 45.9, 60.6, 67.0, and 84.5° (2θ). In contrast, among the deposited metals, only RuO_2_ was detectable, with peaks at 28.1, 35.1, 54.5, and 58.2° (2θ). It should be noted that the form of alumina detected from XRD was Al_2.667_O_4_ due to the nonstoichiometric spinel-type structure of Al_2_O_3_. The scans clearly show that the relative intensity of RuO_2_ peaks increases with the Ru:Fe ratio, reaching a maximum at 100% Ru, confirming that the intended variation in Ru:Fe ratios was achieved in the catalysts.

**Figure 1:**
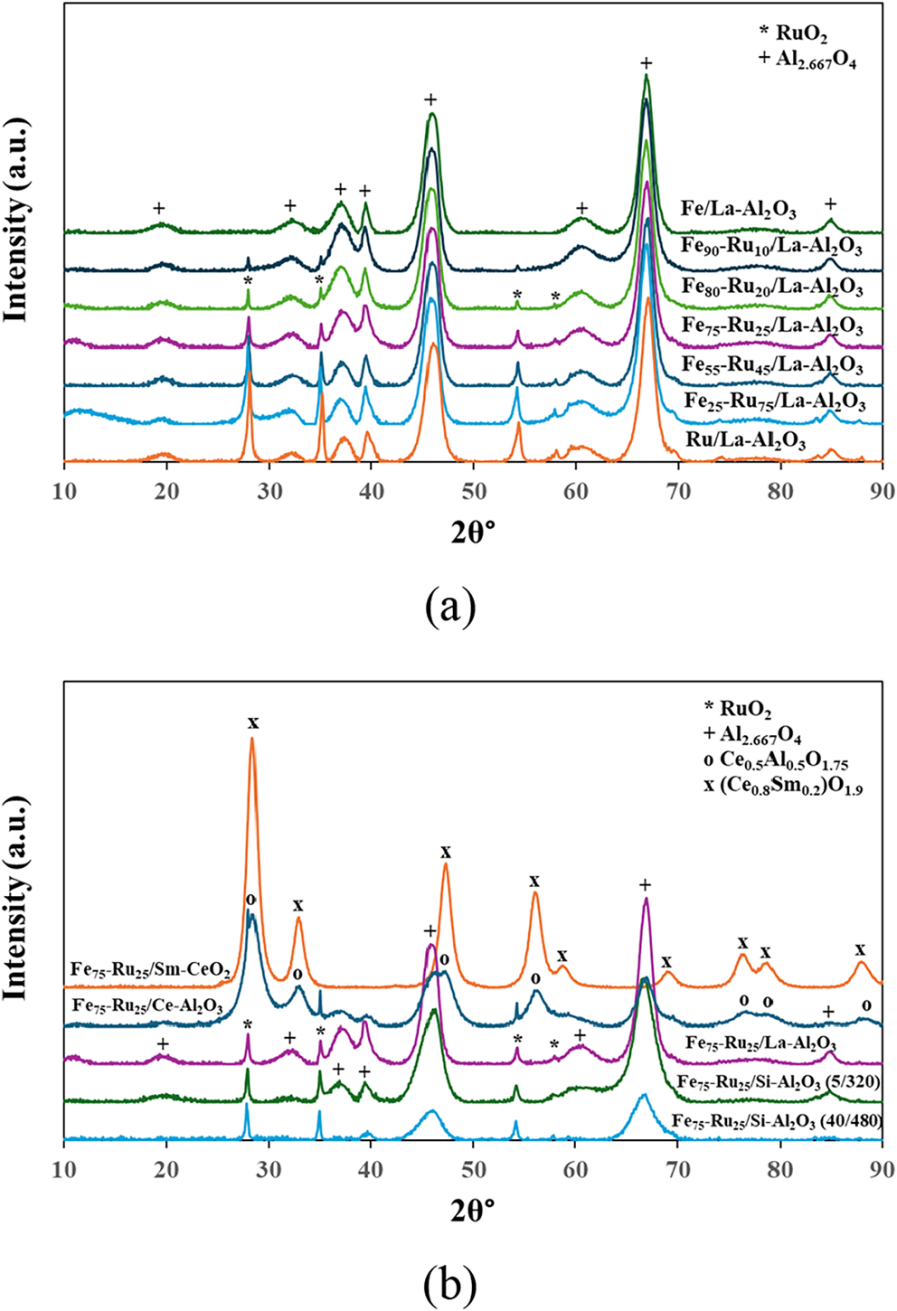
XRD of (a) 2 wt % Fe_*x*_-Ru_*y*_/La–Al_2_O_3_ (*x* = 0, 25, 55, 75, 80, 90, 100; *y* = 0, 10, 20, 25, 45, 75, 100) catalysts and (b) 2 wt % Fe_75_–Ru_25_ on various supports.

Fe_2_O_3_/Fe_3_O_4_ phases are undetectable in the XRD patterns, likely due to their presence as very small and highly dispersed particles, as confirmed by TEM analysis. Furthermore, the low Fe loading (0–2 wt %) combined with high dispersion places these species below the detection limit of XRD, which typically requires crystalline domains above ∼ 3–5 wt % and several nanometers in size. Previous studies have reported that Fe species at similar loadings are difficult to detect by XRD but can be confirmed by complementary techniques such as XPS and H_2_-TPR.^8,28^ Additionally, the use of a Cu Kα radiation source can further reduce sensitivity for light elements, contributing to the absence of Fe-related peaks under these conditions.^8,29^

Figure 1b shows the Fe_75_–Ru_25_ catalysts on various support materials. No Fe_2_O_3_/Fe_3_O_4_ phases were detectable. RuO_2_ was detectable at comparable levels on both La–Al_2_O_3_ and Si–Al_2_O_3_ supports, indicating that the targeted deposited metal ratio was successfully achieved. Alumina (Al_2.667_O_4_) was also the predominant crystalline phase for these supports; however, the peak intensity decreased with the increased presence of Si for the Si–Al_2_O_3_ supports. When ceria is present in the alumina support, Ce–Al_2_O_3_, (indicated as Ce_0.5_Al_0.5_O_1.75_), characteristic peaks were observed at 28.5, 33.2, 47.3, 56.2, 76.5, 79.0 and 88.1 2θ°, for corresponding presence of Ce, as well as characteristic peaks at 19.5, 37.2, 39.6, 45.9, 60.6, 67.0 and 84.5 2θ° for corresponding presence of alumina. RuO_2_ is present (for both Ce–Al_2_O_3_ and Sm-CeO_2_), however, due to some overlap with the crystalline structures of RuO_2_ and CeO_2_, some characteristic peaks (28.1 and 58.2 2θ°) are not detectable. For the peaks at 35.1 and 54.5 2θ°, they are detectable on Ce–Al_2_O_3_; however, it is possible that due to the strong metal–support interaction with Sm-CeO_2_, these are not detected.

Selected catalysts were imaged using TEM (Figure 2) before and after catalytic testing or aging to evaluate changes in the morphology and dispersion of the nanoparticles. Fe_75_–Ru_25_/La–Al_2_O_3_ and Fe_75_–Ru_25_/Ce–Al_2_O_3_ were characterized after the full thermal cycle and subsequent 60-h stability tests (i.e., aging), while Fe_75_–Ru_25_/Sm-CeO_2_ was characterized after only the 3-cycle thermal stability test (i.e., testing).

**Figure 2:**
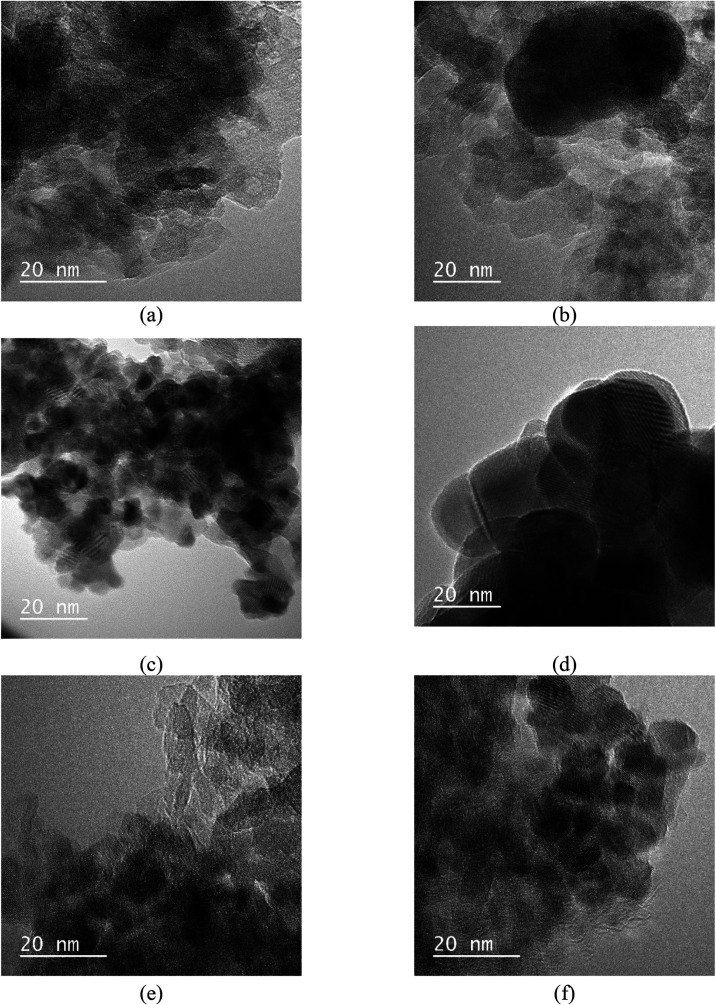
TEM images of the Fe_75_–Ru_25_/La–Al_2_O_3_ (a) before (average particle size = 4.6 nm) and (b) after catalytic testing and aging (average particle size = 22.1 nm); Fe_75_–Ru_25_/Sm-CeO_2_ (c) before (average particle size = 3.9 nm) and (d) after catalytic testing (average particle size = 24.2 nm); Fe_75_–Ru_25_/Ce–Al_2_O_3_ (e) before (average particle size = 4.2 nm) and (f) after catalytic testing and aging (average particle size = 6.4 nm).

Overall, the fresh catalysts (Figure 2a,c,e) show uniformly dispersed nanoparticles with minimal agglomeration. The average particle size was calculated to be 4.6 nm for Fe_75_–Ru_25_/La–Al_2_O_3_, 4.2 nm for Fe_75_–Ru_25_/Ce–Al_2_O_3_, and 3.9 nm for Fe_75_–Ru_25_/Sm-CeO_2_. There is some lattice fringes observed which indicate the crystallinity of the bimetallic clusters of the Fe/Ru nanoparticles.

After testing and aging (Figure 2b,d,f), particle growth and agglomeration became evident for all samples, but with clear differences between supports. The most pronounced coalescence occurred for Fe_75_–Ru_25_/Sm-CeO_2_ (Figure 2d), where large, clustered particles formed (average particle size: 24.2 nm), indicating weaker stabilization by this support. Similarly, Fe_75_–Ru_25_/La–Al_2_O_3_ (Figure 2b) showed some particle growth (average particle size: 22.1 nm), however, with many smaller nanoparticles still relatively well-dispersed, improved resistance to sintering compared with the Sm-CeO_2_support. Fe_75_–Ru_25_/Ce–Al_2_O_3_ (Figure 2f) exhibited the most stable behavior, with some agglomeration but less extensive clustering than on the other supports, with an average particle size of 6.4 nm.

This gradation in agglomeration highlights the role of the support in governing nanoparticle stability, with Ce–Al_2_O_3_ providing the strongest stabilization and Sm-CeO_2_ the weakest. The observed particle growth and loss of dispersion likely contributed to the decline in catalytic activity during aging (Figure 8b).

Figure 3 shows the H_2_-TPR results of all the fresh catalysts investigated in this study, as well as select catalysts postreaction. In all cases for the fresh catalysts, RuO_2_ exhibited reduction at a lower temperature (between 150 and 300 °C) occurring in a stepwise manner, suggesting a range of particle sizes. Fe_2_O_3_/Fe_3_O_4_ reduction (>500 °C) appeared as a broader peak, suggesting the presence of highly dispersed particles.

**Figure 3:**
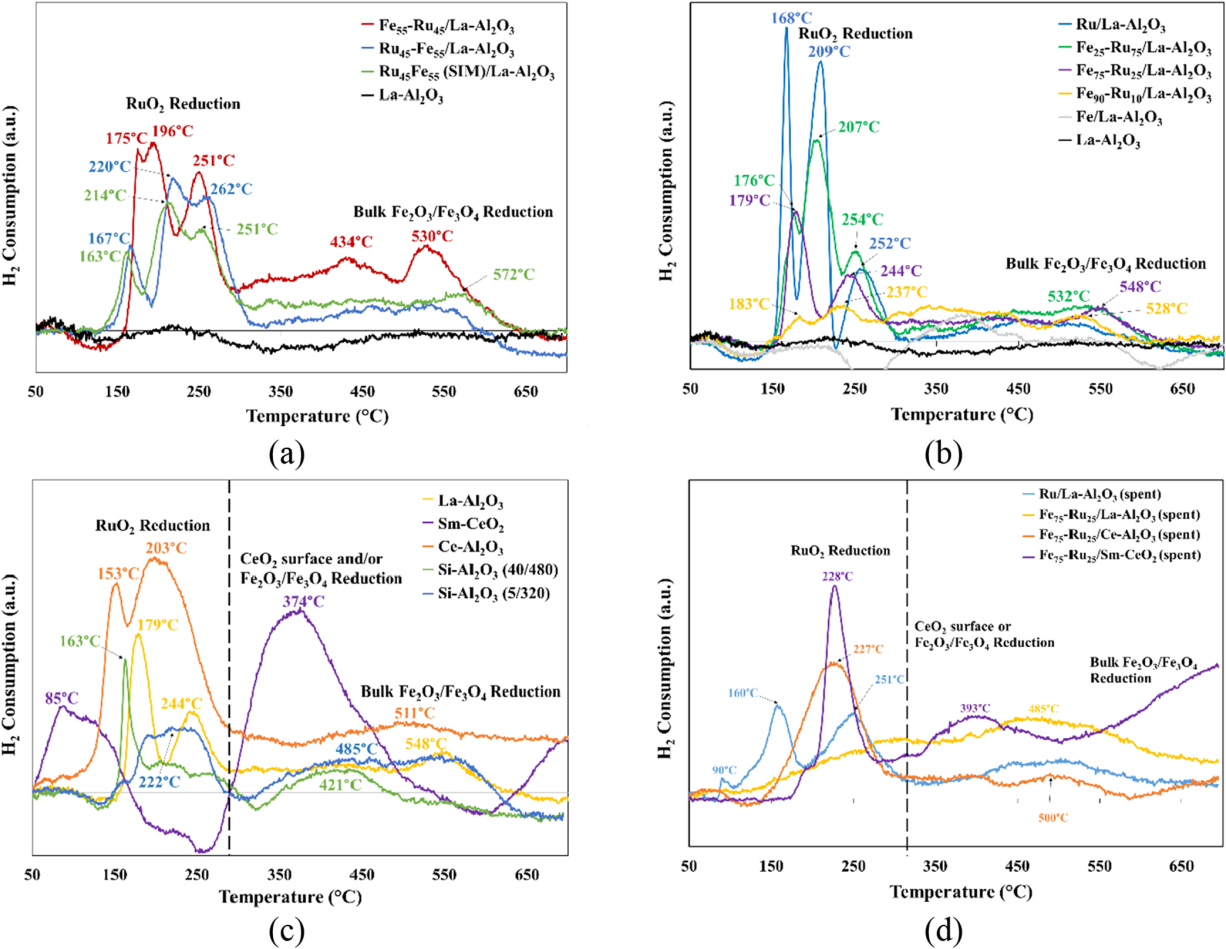
H_2_-TPR profiles of (a) catalysts synthesized with different Fe–Ru deposition approaches on La–Al_2_O_3_, compared to pure La–Al_2_O_3_, before testing (b) Fe_*x*_-Ru_*y*_/La–Al_2_O_3_ (*x* = 0, 25, 75, 90, 100; *y* = 0, 10, 25, 75, 100) catalysts and pure La–Al_2_O_3_, before testing; (c) Fe_75_–Ru_25_ prepared using various support materials, before testing; and (d) select supported Fe_75_–Ru_25_ catalysts, after testing.

From Figure 3a and H_2_ consumption data in Table 2, it was observed that the deposition order significantly influenced the H_2_ consumption for both RuO_2_ and Fe_2_O_3_/Fe_3_O_4_ active sites. The RuO_2_ peaks for both Fe_55_Ru_45_(SIM)/La–Al_2_O_3_ and Ru_45_–Fe_55_/La–Al_2_O_3_ showed a similar multipeak reduction trend (total H_2_ consumption = 7.16 and 8.25 μmol H_2_, respectively). In contrast, Fe_55_–Ru_45_/La–Al_2_O_3_, prepared by adding Fe last to the support showed a different reduction pattern, even though the total H_2_ consumption (8.08 μmol H_2_) remained comparable to the other two catalysts. This shift in RuO_2_ reduction profile for Fe_55_–Ru_45_/La–Al_2_O_3_ suggests a significant change in the distribution and accessibility of RuO_2_ active sites. Furthermore, Fe_2_O_3_/Fe_3_O_4_ reduction (at 434 and 530 °C) was the most prominent for Fe_55_–Ru_45_/La–Al_2_O_3_, with a total H_2_ consumption of 1.90 μmol H_2_. This suggests that depositing Fe after Ru increases the number of reducible Fe^2+^ sites and improves their dispersion. For Ru_45_–Fe_55_/La–Al_2_O_3_, no quantifiable Fe oxide related H_2_ consumption was observed, indicating no significant amount of available Fe sites. The availability of both metal active sites is important as there are two main mechanisms that can occur for the RWGS reaction over Fe and Ru sites – redox and associative pathways. Fe sites are known to enhance adsorption and activation of CO_2_ which favors the redox reaction pathway to directly dissociate CO_2_ to CO and O*. On the other hand, Ru sites are known to facilitate both the dissociation of H_2_ and CO_2_ which is referred to as H-assisted CO_2_ dissociation.^30,31^

**Table 2:** Summary of H_2_-TPR Peaks and Corresponding H_2_ Consumption of Catalysts Synthesized with Different Fe–Ru Deposition Approaches on La–Al_2_O_3_, before Testing^a^

	**peak maxima position (°C)**					**H**_**2**_ **consumption (μmol)**				
**catalyst**	**RuO**_**2**_			**bulk** **Fe**_**2**_ **O**_**3**_ **/Fe**_**3**_ **O**_**4**_		**RuO**_**2**_			**bulk** Fe_**2**_ **O**_**3**_ **/Fe**_**3**_ **O**_**4**_	
Ru_45_–Fe_55_/La–Al_2_O_3_	167	220	262	NQ		1.38	3.53	3.34	NQ	
Ru_45_Fe_55_(SIM)/La–Al_2_O_3_	163	214	251	572		0.71	3.93	2.52	0.90	
Fe_55_–Ru_45_/La–Al_2_O_3_	175	196	251	434	530	1.46	3.48	3.14	0.65	1.25

a NQ = not quantifiable.

Similarly, as shown in Figure 3b and Table 3, varying the Fe:Ru ratio had minimal effect on the overall reduction temperatures, but the shape and intensity of the peaks were significantly affected. There was an obvious decline in total H_2_ consumption for the RuO_2_ sites (from 12.02 μmol H_2_ to not quantifiable) with increasing Fe content, while there seemed to be an optimal H_2_ consumption for Fe_2_O_3_/Fe_3_O_4_ reduction (2.27 μmol H_2_) for the ratio of 75% Fe and 25% Ru. These findings align with catalytic testing (shown in Figure 7a), where coreduction of both metals correlated with improved CO_2_ conversion and CO selectivity in the RWGS reaction.

**Table 3:** Summary of H_2_-TPR Peaks and Corresponding H_2_ Consumption of Fe_*x*_-Ru_*y*_/La–Al_2_O_3_ (*x* = 0, 25, 75, 90, 100; *y* = 0, 10, 25, 75, 100) Catalysts and Pure La–Al_2_O_3_, before Testing^a^

	**peak maxima position (°C)**					**H**_**2**_ **consumption (μmol)**			
**catalyst**	**RuO**_**2**_			**bulk** **Fe**_**2**_ **O**_**3**_ **/Fe**_**3**_ **O**_**4**_	**RuO**_**2**_				**bulk** **Fe**_**2**_ **O**_**3**_ **/Fe**_**3**_ **O**_**4**_
Ru/La–Al_2_O_3_	168	209	252	N/A	3.14		6.10	2.78	N/A
Fe_25_–Ru_75_/La–Al_2_O_3_	176	207	254	532	2.11		6.71	2.78	1.03
Fe_75_–Ru_25_/La–Al_2_O_3_	179		244	548	2.95			1.49	2.27
Fe_90_-Ru_10_/La–Al_2_O_3_	183		237	528	0.21			0.52	0.73
Fe/La–Al_2_O_3_	NQ	NQ	NQ	NQ	NQ		NQ	NQ	NQ

a NQ = not quantifiable; N/A = not applicable.

The impact of the interaction with the support material on the reducibility of the metal catalysts was more significant than either Fe:Ru ratio or deposition order, as shown in Figure 3c and Table 4. For the doped Al_2_O_3_ support materials (La–Al_2_O_3_, Ce–Al_2_O_3_, Si–Al_2_O_3_ (40/480) and Si–Al_2_O_3_ (5/320)), RuO_2_ was reduced in a stepwise manner. However, the presence of Si changed the two distinct steps into a broader peak, with the higher Si present (Si–Al_2_O_3_ (5/320)) diminishing the distinct peaks completely into one broad peak (∼210 °C). The intensity of the RuO_2_ peaks also decreased significantly between Ce–Al_2_O_3_ (16.69 μmol H_2_) and the other doped-alumina supported catalyst, indicating a significant decrease in available active RuO_2_ sites. The catalysts supported on La–Al_2_O_3_ (4.44 μmol H_2_), Si–Al_2_O_3_ (40/480) (4.77 μmol H_2_) and Si–Al_2_O_3_ (5/320) (5.02 μmol H_2_) showed similar H_2_ consumption, indicating a similar amount of RuO_2_ sites available; however, the shape of the profiles indicate a difference in particle size and distribution. This observed trend of decreasing H_2_ consumption and changing in TPR profiles correlates to the decrease in CO_2_ conversion (shown in Figure 7d), which indicates the importance of the availability and reducibility of the RuO_2_ active site.

**Table 4:** Summary of H_2_-TPR Peaks and Corresponding H_2_ Consumption of Fe_75_–Ru_25_ Prepared Using Various Support Materials, before Testing^a^

**catalyst**	**peak maxima position (°C)**				**H**_**2**_ **consumption (μmol)**			
	**RuO**_**2**_		**surface CeO**_**2**_ **and/or** **Fe**_**2**_ **O**_**3**_ **/Fe**_**3**_ **O**_**4**_	**bulk** **Fe**_**2**_ **O**_**3**_ **/Fe**_**3**_ **O**_**4**_	**RuO**_**2**_		**surface CeO**_**2**_ **and/or** **Fe**_**2**_ **O**_**3**_ **/Fe**_**3**_ **O**_**4**_	**bulk** **Fe**_**2**_ **O**_**3**_ **/Fe**_**3**_ **O**_**4**_
Fe_75_–Ru_25_/La–Al_2_O_3_	179	244	N/A	548	2.95	1.49	N/A	2.27
Fe_75_–Ru_25_/Sm-CeO_2_	85		374	694	7.47		23.30	NQ
Fe_75_–Ru_25_/Ce–Al_2_O_3_	153	203^b^		511	4.16	12.53		1.55
Fe_75_–Ru_25_/Si–Al_2_O_3_ (40/480)	163^c^		N/A	421	4.77		N/A	2.90
Fe_75_–Ru_25_/Si–Al_2_O_3_ (5/320)	222^c^		N/A	485^c^	5.02		N/A	7.70

a NQ = not quantifiable; N/A = not applicable.

b Possible contribution to H_2_ consumption by CeO_2_ in support.

c Taken as a representative temperature for the broad peak.

For the Sm-CeO_2_ supported catalyst, the reduction of RuO_2_ occurred at a much lower temperature, shown by the broad peak centered near 100 °C. The H_2_ consumption (7.47 μmol H_2_) was lower than the Ce–Al_2_O_3_ supported catalysts, possibly due to the strong interaction with the support that may have reduced the available active RuO_2_ sites. Doping with Sm increases oxygen vacancies in the CeO_2_ lattice, which can facilitate hydrogen spillover and electron transfer to RuO_2_.^32,33^ A subsequent negative H_2_ consumption was observed, near 250 °C, likely indicating hydrogen retention by the highly reducible support.^32,33^ This H_2_ retention is important to note as it has a significant impact on the overall catalytic performance (shown in Figure 7c) and the availability of H_2_ to reduce CO_2_ in the RWGS reaction. Sm-CeO_2_ also shows a large and broad peak at 374 °C (23.30 μmol H_2_) which was attributed to either surface CeO_2_ reduction and/or early Fe_2_O_3_/Fe_3_O_4_ reduction due to highly dispersed nanoparticles. The peak above 650 °C (not quantifiable due to being out of the temperature range) was attributed to bulk Fe_2_O_3_/Fe_3_O_4_ reduction and could be a factor in the high CO_2_ conversion at higher temperatures (>650 °C) (shown in Figure 7d).

Overall, for the different supports, there was a wide variation in the reduction temperature for Fe_2_O_3_/Fe_3_O_4_, indicating a significant influence of metal–support interactions, as well as very broad peaks indicating highly dispersed particles. As stated previously, it is important that both active metals are reducible within the operating temperature range of the RWGS reaction for high CO_2_ conversion and CO selectivity. It is evident that Fe_75_–Ru_25_/Ce–Al_2_O_3_ shows significant H_2_ consumption for both RuO_2_ and Fe_2_O_3_/Fe_3_O_4_, which was a contributing factor in its high catalytic performance (Figure 7c).

The H_2_-TPR profiles of selected spent catalysts are shown in Figure 3d, with the corresponding H_2_ consumption of each catalyst summarized in Table 5. It is evident that the overall profiles changed after testing and/or aging. In comparison to the fresh catalyst, the RuO_2_ reduction peaks for Ru/La–Al_2_O_3_ shifted to slightly lower temperatures and exhibited much lower overall H_2_ consumption (12.02 μmol H_2_ to 5.27 μmol H_2_), indicating partial sintering or changes in Ru dispersion during reaction, resulting in a loss of active RuO_2_ sites. The Fe_75_–Ru_25_/La–Al_2_O_3_ catalyst showed a significant loss in RuO_2_ active sites (i.e., not quantifiable). Its TPR profile displays a very broad overall peak, making it difficult to identify the RuO_2_ contribution. Similarly, the peak associated with Fe_2_O_3_/Fe_3_O_4_ reduction shifted to a lower temperature, and H_2_ consumption was reduced from 2.27 μmol H_2_ to 0.45 μmol H_2_. In contrast, the Fe_75_–Ru_25_/Ce–Al_2_O_3_ catalyst showed an increase in temperature for the RuO_2_ peak (from 153/203 to 228 °C) as well as a change from two-step reduction to a single step compared to the fresh catalyst. A similar bulk Fe_2_O_3_/Fe_3_O_4_ reduction peak was observed around 500 °C. An overall decrease in H_2_ consumption for both RuO_2_ and Fe_2_O_3_/Fe_3_O_4_ active sites was observed, indicating a loss of available active sites for the reaction to occur.

**Table 5:** Summary of H_2_-TPR Peaks and Corresponding H_2_ Consumption of Select Supported Fe_75_–Ru_25_ Catalysts, Comparing before and After Testing^a^,^c^

		**peak maxima position (°C)**					**H**_**2**_ **consumption (μmol)**				
**catalyst**		**RuO**_**2**_			**surface CeO**_**2**_ **and/or Fe**_**2**_ **O**_**3**_ **/ Fe**_**3**_ **O**_**4**_	**bulk Fe**_**2**_ **O**_**3**_ **/Fe**_**3**_ **O**_**4**_	**RuO**_**2**_			**surface CeO**_**2**_ **and/or Fe**_**2**_ **O**_**3**_ **/Fe**_**3**_ **O**_**4**_	**bulk Fe**_**2**_ **O**_**3**_ **/Fe**_**3**_ **O**_**4**_
Ru/La–Al_2_O_3_	before	168	209	252	N/A	N/A	3.14	6.10	2.78	N/A	N/A
	after	90	160	251	N/A	N/A	0.18	2.28	2.81	N/A	N/A
Fe_75_–Ru_25_/La–Al_2_O_3_	before	179		244	N/A	548	2.95		1.49	N/A	2.27
	after	NQ		NQ	N/A	485	NQ		NQ	N/A	0.45
Fe_75_–Ru_25_/Sm-CeO_2_	before	85			374	694		7.47		23.30	NQ
	after	228			393	694		6.03		2.80	NQ
Fe_75_–Ru_25_/Ce–Al_2_O_3_	before	153		203^b^		511	4.16		12.53		1.55
	after	227^b^				500	9.34				0.87

a NQ = not quantifiable; N/A = not applicable.

b Possible contribution to H_2_ consumption by CeO_2_ in support.

c Taken as a representative temperature for the broad peak.

The Fe_75_–Ru_25_/Sm-CeO_2_ catalyst showed the most significant change in its TPR profile, after testing, the RuO_2_ shifted to a higher temperature (228 °C) and the peak changed from a broad to sharp peak (6.03 μmol H_2_), indicating an increase in Ru particle homogeneity on the surface. This resulting homogeneity could be a contributing factor to the significant increase in catalytic activity observed at temperatures above 550 °C. There was no observed temperature shift for the combined surface CeO_2_ and Fe_2_O_3_/Fe_3_O_4_ reduction; however, the peak intensity decreased (2.80 μmol H_2_) and became very broad. The H_2_ consumption for this peak decreased substantially, indicating severe restructuring and loss of reducible Fe_2_O_3_/Fe_3_O_4_ or CeO_2_ species.

Overall, it is evident that significant structural changes and loss of active sites occurred during the reaction for all catalysts but to different extents, reflecting the thermal stability and metal–support interactions of these catalysts. Among the samples, Fe_75_–Ru_25_/Ce–Al_2_O_3_ showed the least amount of change, which could explain its higher overall performance.

XPS was employed to investigate the surface chemical composition and oxidation states of the elements present in selected catalysts, providing insights into the electronic environment and surface interactions relevant to their catalytic performance (Figure 4). For all catalysts, evident in their respective wide range scans (Figure (a), (d), (f)), a high oxygen content was present on the surface as well as the corresponding metal from the support materials (i.e., Al, Ce, La, Sm). Note, the high C 1s presence was attributed to adventitious carbon. Additionally, due to the low Ru loading (0.5 wt %), the Ru 2p region exhibited a very weak signal with poor signal-to-noise ratio, making it unsuitable for reliable identification or quantification. Therefore, Ru analysis was performed in the Ru 3d region, despite its partial overlap with C 1s, using careful fitting constraints to minimize interference.

**Figure 4:**
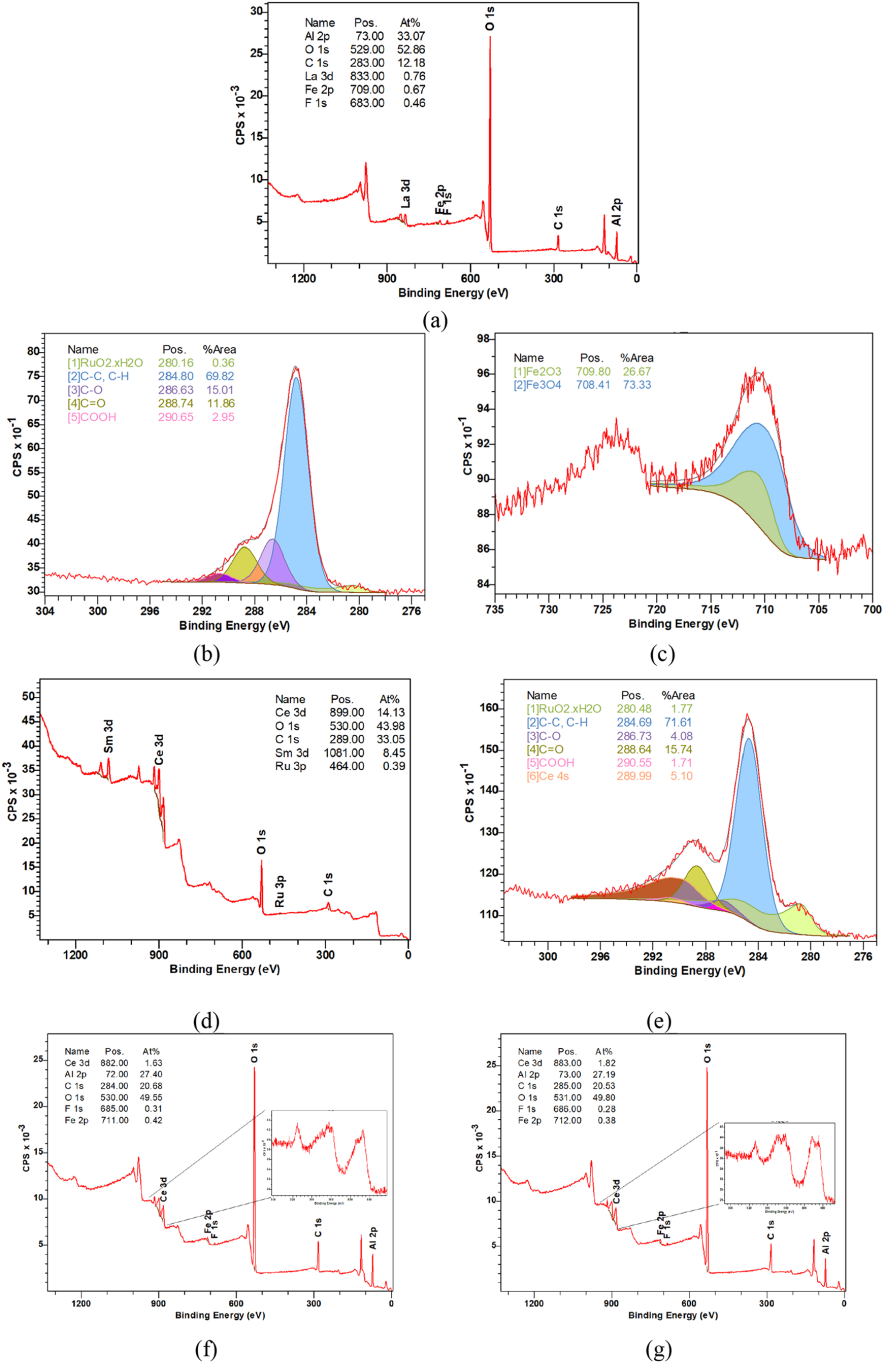
XPS analysis of (a) wide range scan, (b) high resolution C­(1s), (c) high resolution Fe­(2p) of Fe_75_–Ru_25_/La–Al_2_O_3_ (before catalytic testing); (d) wide range scan, (e) high resolution C­(1s) of Fe_75_–Ru_25_/Sm-CeO_2_ (before catalytic testing) and wide range scan of (f) Fe_75_–Ru_25_/Ce–Al_2_O_3_ (before catalytic testing) and (g) Fe_75_–Ru_25_/Ce–Al_2_O_3_ (after catalytic testing).

For the La–Al_2_O_3_-supported catalyst (Figure 4a–c), Ru^4+^ (Figure 4b), Fe^3+^ and Fe^2+^ (Figure 4c) states were observed, however, they are observed in very low quantities because of high dispersion and low metal loading. In Figure 4c, Fe^2+^ (∼73%) is shown as being the dominant iron species indicating that the fresh catalyst has a mixture of Fe^3+^ and Fe^2+^ present at the surface of the catalyst. This mixed oxidation state is beneficial for the RWGS reaction as Fe^2+^ can facilitate oxygen mobility and electron transfer which help reduce the CO_2_ at the surface.^34^

Among all of the oxidation states of ruthenium, Ru^4+^ was observed to be the prominent species, for both Fe_75_–Ru_25_/La–Al_2_O_3_ (Figure 4b) and Fe_75_–Ru_25_/Sm-CeO_2_ (Figure 4e). The presence of this species has been shown to promote the dissociation of CO_2_ on the catalyst surface and the formation of water.^31^

Similar results for Ru^4+^, Fe^3+^ and Fe^3+^ were observed for the Fe_75_–Ru_25_/Ce–Al_2_O_3_ catalyst. In this case, the primary purpose of characterization was to compare the catalyst before and after testing (Figure 4f,g to gain an insight into its stability (as reflected in Figure 8)). Both fresh and tested samples showed a mixture of Ce^4+^ and Ce^3+^, with a higher Ce^3+^ content in the catalyst after testing. While the presence of Ce­(III) promotes oxygen vacancies and facilitates CO_2_ activation, too much can reduce the support material’s ability to reoxidize, which is essential for sustained RWGS catalytic activity.^35^

The role of oxygen vacancies in promoting SMSI has been widely discussed in the literature. Although O 1s XPS is often employed to estimate oxygen vacancy concentrations,^36^ recent critical evaluations have highlighted significant limitations of this approach. Easton and Morgan^37^ demonstrated that O 1s peak deconvolution cannot uniquely identify oxygen vacancies due to overlapping contributions from hydroxyl groups, adsorbed species, and surface heterogeneity. Similarly, theoretical studies by Posada-Borbón et al.^38^ indicate that O 1s binding energy shifts associated with vacancies are minor and easily confounded by other surface species. In light of these findings, the authors refrained from making quantitative claims based on O 1s XPS in this work. Instead, indirect evidence of SMSI is provided through enhanced reducibility observed in TPR profiles, which is consistent with strong metal–support interactions.

### 3.2 Catalytic Performance

The catalytic performance of the three bimetallic catalysts, Ru_45_Fe_55_(SIM)/La–Al_2_O_3_, Fe_55_–Ru_45_/La–Al_2_O_3_, and Ru_45_–Fe_55_/La–Al_2_O_3_, was evaluated under reaction conditions using an H_2_:CO_2_ ratio of 1:1 (diluted in N_2_). As shown in Figure 5a, the catalyst prepared by depositing Ru first, followed by Fe (Fe_55_–Ru_45_/La–Al_2_O_3_) exhibited the highest CO_2_ conversion among the three approaches, particularly at lower temperatures. These results (i.e., Ru deposited first, then Fe) were attributed to the role of Ru acting as a stabilizer for Fe by creating a protective boundary around the Fe nanoparticles to prevent aggregation.^24,39^ When Ru is deposited first, it forms small, well-dispersed nanoparticles that act as nucleation sites for Fe species, promoting intimate Fe–Ru contact. This configuration facilitates electronic modification of Fe through charge transfer, enhancing CO_2_ activation and favoring RWGS. Additionally, Ru helps inhibit Fe particle sintering during high-temperature operation by creating a physical boundary, thereby maintaining high surface area and active site accessibility^40,41^

**Figure 5:**
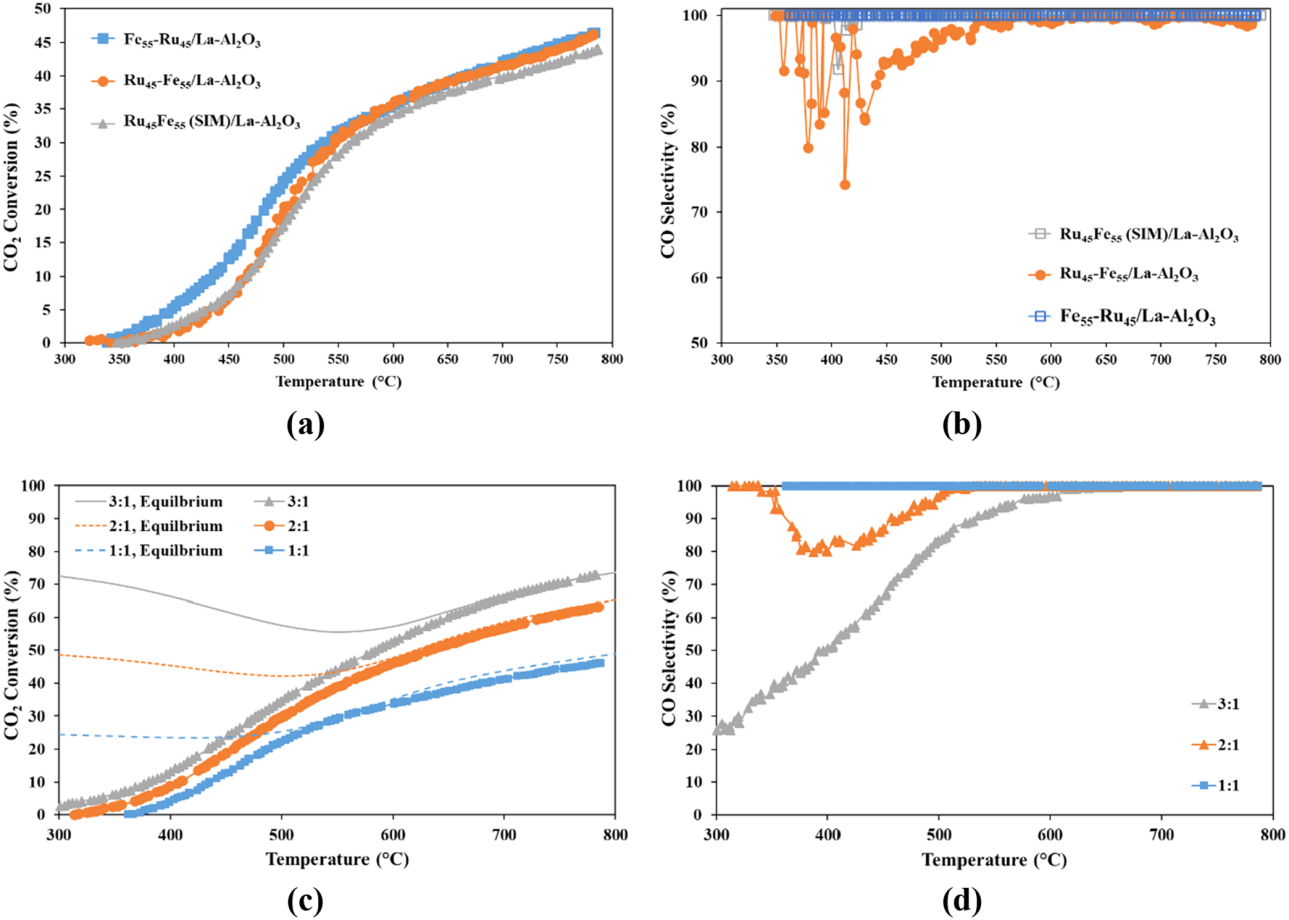
Effect of deposition order of 2 wt % Ru and Fe on La–Al_2_O_3_ (H_2_/CO_2_ = 1:1) on (a) CO_2_ conversion and (b)­CO selectivity; and H_2_:CO_2_ ratio on (c) CO_2_ conversion and (d) CO selectivity at different temperatures of Fe_55_–Ru_45_/La–Al_2_O_3_ catalyst (Testing Conditions: total flow rate = 400 mL·min^–1^, WHSV = 135 h^–1^).

As shown in Figure 5b, the CO selectivity was close to 100% for both catalysts that Fe was deposited after (Fe_55_–Ru_45_/La–Al_2_O_3_) or simultaneously with Ru (Ru_45_Fe_55_(SIM)/La–Al_2_O_3_), while, at low temperatures (<550 °C), the CO selectivity varied between 75 and 100% for the catalyst with Ru deposited after Fe (Ru_45_–Fe_55_/La–Al_2_O_3_). It is well established that Ru is selective toward methanation.^25,26^ These results demonstrate that the availability of Fe sites, as well as the synergistic effect of Fe combined with Ru are important for CO selectivity.

Furthermore, as previously shown through H_2_-TPR analysis (Figure 3a), the Fe_55_–Ru_45_/La–Al_2_O_3_ catalyst had the highest H_2_ consumption, especially for Fe_2_O_3_/Fe_3_O_4_, showing the presence of available, reducible active sites. The availability of these reducible active sites is a desirable attribute of a catalyst for the RWGS reaction. Based on these results, the catalyst with Ru-first/Fe-second deposition sequence was studied further in this work.

Figure 5c shows the effect on CO_2_ conversion of increasing the H_2_ concentration from a 1:1 to 3:1 ratio for the Fe_55_–Ru_45_/La–Al_2_O_3_ catalyst. The higher ratio of H_2_:CO_2_, 3:1, resulted in higher conversion, due to the availability of more H_2_ to reduce CO_2_ to CO. However, since H_2_ is one of the significant cost factors in implementing the RWGS reaction at larger scale, a ratio of 1:1 was chosen for the remaining experiments as the performance was deemed relatively close to equilibrium, especially at higher temperatures (i.e., >500 °C). These results also indicated that activity at lower temperatures (≤500 °C) could potentially be improved by varying the Fe:Ru ratio and support materials, which were further investigated in this study.

To obtain the equilibrium data shown in Figure 5b, a Gibbs reactor model was implemented in Aspen HYSYS. The reactor minimizes the total Gibbs free energy of the system for the specified feed composition and operating conditions. The calculations included the RWGS reaction (eq 1) and methanation reactions (eqs 2 and 3) to represent the combined equilibrium behavior. The feed ratios (1:1, 2:1 and 3:1 H_2_:CO_2_) were applied at a pressure of 1 bar over a temperature range of 300–800 °C.

The CO selectivity trends for the different H_2_:CO_2_ ratios are shown in Figure 5d. As observed, the H_2_:CO_2_ ratio strongly influences CO selectivity in the RWGS reaction because it affects the equilibrium and competitive side reactions.^42^ At higher H_2_ ratios (e.g., 3:1), excess hydrogen promotes methanation, reducing CO selectivity at lower temperatures. As temperature increases, methanation becomes less favorable, and CO selectivity rises. At moderate ratios (2:1), methanation still occurs but to a lesser extent, leading to intermediate selectivity trends. In contrast, the 1:1 ratio maintains nearly 100% CO selectivity across all temperatures because the stoichiometry limits hydrogen availability for methanation, favoring CO formation through RWGS.^43^ Due to these results, a H_2_:CO_2_ ratio of 1:1 was chosen for the remaining experiments.

To reduce the overall cost of the catalyst, different Fe:Ru ratios were deposited on the La–Al_2_O_3_ support to determine whether partial substitution of Ru with the less expensive Fe could maintain good catalytic performance. Figure 6a summarizes the CO_2_ conversion of the catalysts at 500 °C with respect to the RWGS reaction equilibrium. The Ru/La–Al_2_O_3_ catalyst is the best performing in terms of CO_2_ conversion (25%). These results can be attributed to Ru being known for its stability and high catalytic activity, as well as the relatively higher surface area compared to other catalysts.^44^ It should be noted, however, that the CO selectivity for Ru/La–Al_2_O_3_ was 87% (i.e., methane formation was observed),^45^ while for the rest of the catalysts, it was approximately 100%. By adding up to 75% Fe, good CO_2_ conversion (22 vs 25% at equilibrium) was still achieved, as well as the desirable increase in CO selectivity (100%), even though a slight decrease in surface area was also observed. However, the decrease in surface area was not the only factor contributing to the decrease in CO_2_ conversion as it remained consistent for the rest of the catalysts. At 80% Fe up to 100% Fe, the CO_2_ conversion decreased significantly. These low conversion results with high amounts of Fe are attributed to the instability of Fe nanoparticles and their tendency to aggregate.^46,47^ Overall, it is evident that the presence of both Fe and Ru have a synergistic effect, that is, Ru stabilizing the Fe nanoparticles and Fe promoting CO desorption, preventing CH_4_ formation by Ru.^24,39^

**Figure 6:**
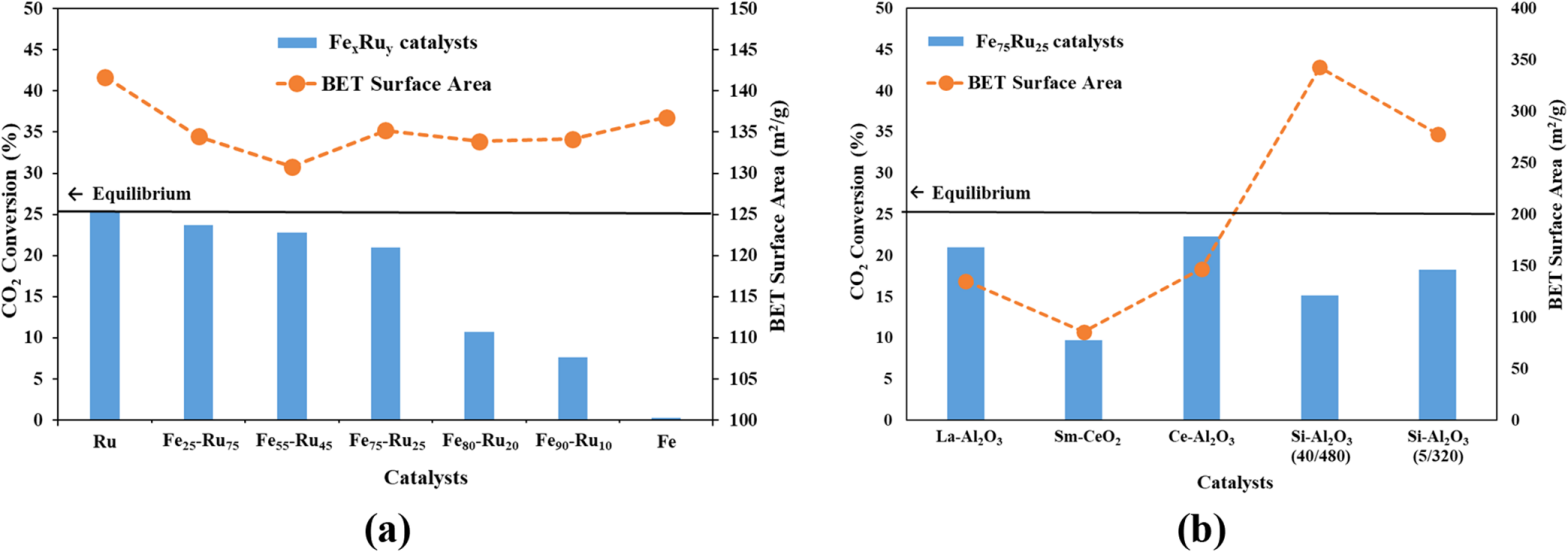
CO_2_ Conversion at 500 °C of (a) 2 wt % Fe_*x*_-Ru_*y*_/La–Al_2_O_3_ (*x* = 0, 25, 55, 75, 80, 90, 100; *y* = 0, 10, 20, 25, 45, 75, 100) and (b) 2 wt % Fe_75_–Ru_25_ on different supports and corresponding BET surface area. (Testing Conditions: H_2_:CO_2_ = 1:1, total flow rate = 400 mL·min^–1^, WHSV = 135 h^–1^).

The effects of various support materials on the Fe_75_–Ru_25_ catalyst were further systematically investigated. The results are shown in Figure 6b. The highest CO_2_ conversion (21 and 22%, respectively) at 500 °C was obtained using the La-doped Al_2_O_3_ and Ce-doped Al_2_O_3_ supports, which correspondingly have similar surface areas. However, as indicated earlier, surface area is not the only factor affecting the catalytic activity. Although the catalysts supported on Si–Al_2_O_3_ exhibited significantly higher surface areas compared to the other supports, this did not translate into higher catalytic activity. In contrast, the low surface area of the Sm-doped ceria support likely contributed to its poor catalytic performance, which was further influenced by strong Fe–Ru/Sm–CeO_2_ metal–support interactions. A similar negative effect of SMSI was demonstrated in some previous studies. Bowker et al.^23^ showed that TiO_2_ can form a layer over Pd nanoparticles or create intermetallic compounds, resulting in reduced active sites. Another study showed that depending on the phase of TiO_2_ used to support Ru particles, the strength of metal–support interaction was altered, changing the catalytic performance.^45^

Figure 7 shows the (a) CO_2_ conversion and (b) CO selectivity trends with varying temperatures for the La–Al_2_O_3_ supported catalysts with different Fe:Ru ratios. As discussed previously, the Ru/La–Al_2_O_3_ catalyst is the best performing in terms of CO_2_ conversion; however, lower CO selectivity was observed for temperatures below 535 °C (Figure 7b). By introducing Fe into the catalyst, there is a clear separation between maintaining high catalytic performance (i.e., close to that of 100% Ru) and the decline in CO_2_ conversion for 80% Fe or higher. In contrast to the Ru/La–Al_2_O_3_ catalyst, the CO selectivity approached 100% as the content of Fe was increased (i.e., for 55% or more). Adding Fe provides active sites that favor CO_2_ activation and oxygen removal, while Ru primarily facilitates hydrogen dissociation. This synergistic interaction between Fe and Ru promotes the formation of CO rather than methane by suppressing methanation pathways and stabilizing intermediates associated with CO production.^30,31^

**Figure 7:**
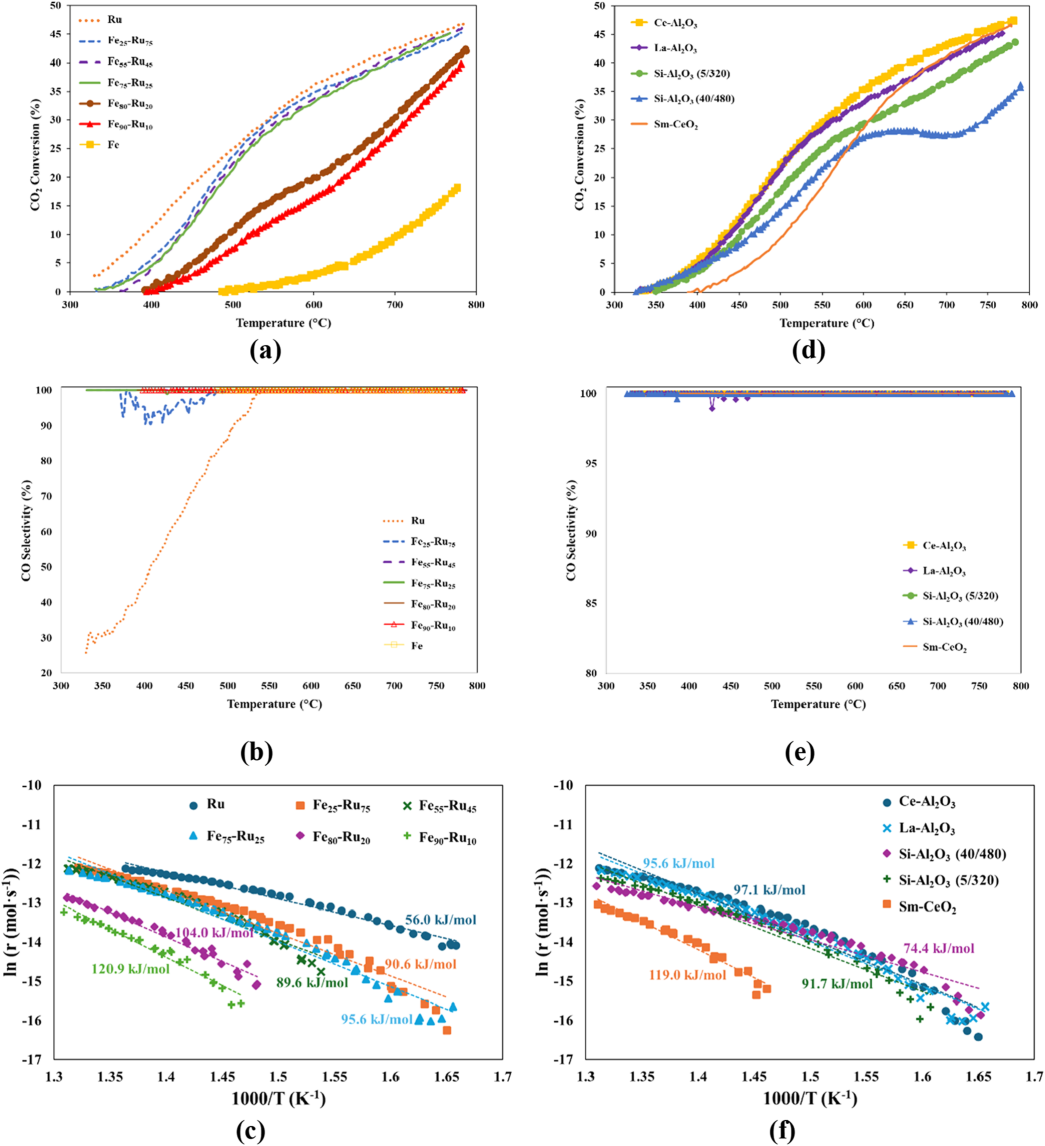
Comparison of catalytic performance at different temperatures (300 to 800 °C) for CO_2_ conversion, CO selectivity and corresponding Arrhenius plots (temperature range from 330 to 490 °C) of (a,b,c) 2 wt % Fe_*x*_-Ru_*y*_/La–Al_2_O_3_ with different Fe:Ru ratios and (d, e, f) Fe_75_–Ru_25_ on different supports (Testing Conditions: H_2_:CO_2_ = 1:1, total flow rate = 400 mL·min^–1^, WHSV = 135 h^–1^).

In addition, the activation energy for the catalysts (330 to 490 °C) was calculated (Figure 7c) and confirmed the distinct superior catalytic performance of Ru/La–Al_2_O_3_ (*E*_a_ = 56.0 kJ·mol^–1^). It should be noted that the activity of Fe only catalyst did not fall within the feasible temperature range to be able to calculate its activation energy. These results also highlight that incorporating 80% Fe or more compromises the synergistic effect with Ru, ultimately reducing catalytic activity. Overall, from these experiments, it was determined that the ratio of Fe:Ru 75:25 (*E*_a_ = 95.6 kJ/mol) was the best one to continue the study on various supports.

The significant variation in apparent activation energies (56.0–120.6 kJ/mol) observed for catalysts with different Ru:Fe ratios on La–Al_2_O_3_ (Figure 7c) highlights the critical role of bimetallic composition in governing RWGS kinetics. Higher Ru content lowers the activation energy by enhancing hydrogen activation and facilitating rapid removal of surface oxygen species, which promotes the CO_2_ dissociation pathway with a lower kinetic barrier. Conversely, Fe-rich catalysts exhibit higher activation energies due to stronger Fe–O interactions and reduced hydrogen activation capability, which stabilize intermediate species and slow oxygen exchange. These trends are supported by TPR analyses (Figure 3b and Table 3), which show that Ru-rich catalysts are more reducible and exhibit distinct surface oxidation states compared to Fe-rich compositions. Thus, the Ru:Fe ratio modulates both the chemical environment and the dominant reaction mechanism, explaining the observed differences in activation energy even on the same support.

The CO_2_ conversion and CO selectivity results obtained for the Fe_75_–Ru_25_ catalyst on five different support materials, namely, La–Al_2_O_3_, Ce–Al_2_O_3_, Si–Al_2_O_3_ (5/320), Si–Al_2_O_3_ (40/480) and Sm-CeO_2_ are shown in Figure 7d,e, respectively. It is evident that the support material did not have a significant effect on the CO selectivity, with all catalysts showing >99% over the range of temperatures. This suggests that CO selectivity in the RWGS reaction is primarily governed by the intrinsic properties of the Fe–Ru active phase rather than the support material. Since the reaction mechanism is dominated by the metal sites responsible for CO_2_ reduction and hydrogen activation, variations in support characteristics (e.g., surface area) have minimal influence on the selectivity toward CO. It was found that the best performing catalyst was Fe_75_–Ru_25_/Ce–Al_2_O_3_, closely followed by Fe_75_–Ru_25_/La–Al_2_O_3_. The higher activity of the Fe_75_–Ru_25_/Ce–Al_2_O_3_ catalyst is attributed to, first, the high dispersion of RuO_2_ and Fe_2_O_3_/Fe_3_O_4_ particles (as observed from H_2_-TPR), as well as the higher surface area that is provided by the Al_2_O_3_-based support. Second, the presence of oxygen vacancies due to the Ce dopant which allows for enhanced CO_2_ adsorption and CO desorption. According to the TPR results (Figure 3c), Fe_75_–Ru_25_/Ce–Al_2_O_3_ and Fe_75_–Ru_25_/La–Al_2_O_3_ showed the highest reducibility of both RuO_2_ and Fe_2_O_3_/Fe_3_O_4_, therefore, this is also a significant contributing factor to the overall catalytic performance. This is an important factor as reduced Ru^0^ sites can efficiently split H_2_ molecules into atomic hydrogen, while reduced Fe^0^ or Fe^2+^ sites interact strongly with CO_2_, making it more reactive. Having both these sites in close vicinity promotes a faster reaction of the H_2_ and CO_2_ to produce CO.^48–51^

Below 600 °C, the Si–Al_2_O_3_ supported catalysts showed better performance than the Sm-CeO_2_ supported catalyst, likely due to the stability from Al_2_O_3_ and reduction of both RuO_2_ and Fe_2_O_3_/Fe_3_O_4_ (as observed from the TPR results). Although Sm-CeO_2_ contains a high concentration of oxygen vacancies, their effect is primarily associated with SMSI and H_2_ spillover from the metal particles to the support (as discussed in the TPR results). This contrast in reduction behavior between doped Al_2_O_3_ and doped CeO_2_ is demonstrated. Above 600 °C, Fe_75_–Ru_25_/Sm-CeO_2_, outperforms the La–Al_2_O_3_ supported catalyst. As was observed from the TPR for Fe_75_–Ru_25_/Sm-CeO_2_, bulk reduction of Fe_2_O_3_/Fe_3_O_4_ occurred at a higher temperature (∼650 °C), corresponding to approximately the inflection point at which the CO_2_ conversion drastically increased. Additionally, it was observed from TPR (Figure 3d) that postreaction a significant restructuring of this catalyst occurred in favor of improved homogeneity of the RuO_2_ active sites, contributing to the drastic increase in catalytic activity at a higher temperature. This observation demonstrates the importance of both metals being reduced to obtain high catalytic performance. This delayed catalytic performance was confirmed by calculating the activation energy (Figure 7d), where the Sm-CeO_2_ supported catalyst showed a significantly higher activation energy (119.0 kJ/mol) compared to all of the other supported catalyst. Although Sm-CeO_2_ exhibits a high concentration of oxygen vacancies that promote strong metal–support interactions (SMSI), these interactions may lead to partial encapsulation of the active metal sites, thereby hindering their accessibility and negatively impacting the catalytic performance in the RWGS reaction.

It was observed that the presence of oxygen vacancies in the support materials affects the interaction between the active surface sites (RuO_2_ and Fe_2_O_3_/Fe_3_O_4_) and the reactants (CO_2_ and H_2_), contributing to the overall catalytic performance, especially at lower temperatures (i.e., < 500 °C). Figure 7f depicts this influence and trend, with the activation energy decreasing in the order: (*E*_a(Sm‑CeO_2_)_ = 119.0 kJ/mol; *E*_a(Ce–Al_2_O_3_)_ = 97.1 kJ/mol; *E*_a(La–Al_2_O_3_)_ = 95.6 kJ/mol; *E*_a(Si–Al_2_O_3_ (5/320))_ = 91.7 kJ/mol; *E*_a(Si–Al_2_O_3_ (40/480))_ = 74.4 kJ/mol). This decrease correlates with the relative concentration of oxygen vacancies across the supports (Sm-CeO_2_ > Ce–Al_2_O_3_ > La–Al_2_O_3_ > Si–Al_2_O_3_ (5/320) > Si–Al_2_O_3_ (40/480)).^19,52^

The stability of selected catalysts was evaluated in two ways: after 3 temperature ramp cycles and at a constant temperature (500 °C) over 60 h of operation (Figure 8). The temperature ramp stability and reproducibility with respect to CO_2_ conversion and CO selectivity of the overall best performing catalyst, Fe_75_–Ru_25_/Ce–Al_2_O_3_, is shown in Figure 8a. The CO selectivity remained close to 100% throughout most cycles; however, a slight decrease was observed below 400 °C during Run 2, Cycle 1. This reduction could be attributed to the inherently low catalytic activity at these temperatures, combined with the initial restructuring of the catalyst. After each cycle, the catalytic performance improved slightly (∼5% CO_2_ conversion). This trend was consistent between the two fresh catalysts. These observations can be attributed to potential redispersion of metal particles (i.e., temperature cycling can break up larger particles into smaller ones), changes in surface composition and morphology that could expose more active sites, and/or changes in the oxidation state of the active metals and supports (i.e., in the case of Ce-containing supports).^53^ As observed, using XPS, after testing, higher Ce­(III) was present in the sample which can enhance oxygen mobility and CO_2_ activation, resulting in higher CO_2_ conversion.

**Figure 8:**
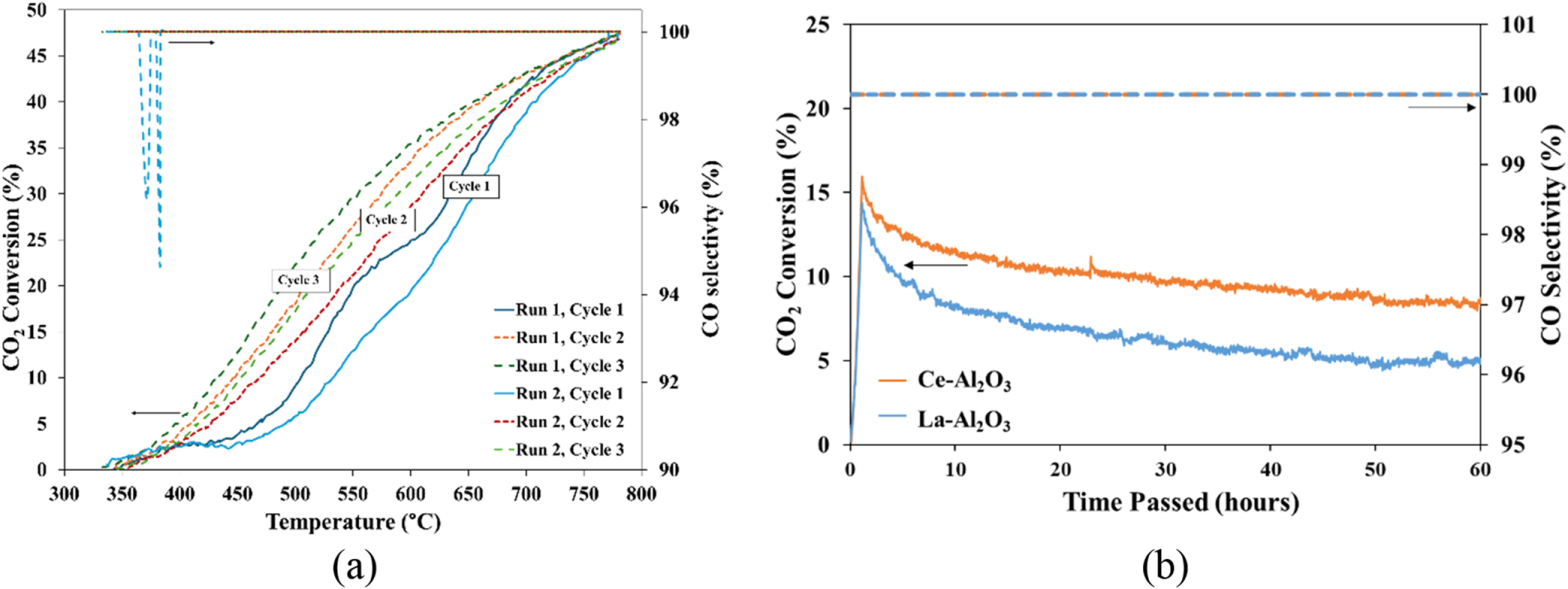
Effect on CO_2_ conversion and CO selectivity of (a) temperature ramp cycles (Cycle 1, 2, 3) for the 2 wt % Ru_75_–Fe_25_/Ce–Al_2_O_3_ catalyst and reproducibility (Run 1 and 2) between two fresh catalysts; and (b) long-term (60 h) exposure at 500 °C for the 2 wt % Ru_75_–Fe_25_/Ce–Al_2_O_3_, and Ru_75_–Fe_25_/La–Al_2_O_3_ catalysts (Testing Conditions: H_2_:CO_2_ 1:1, total flow rate = 400 mL·min^–1^, WHSV = 135 h^–1^).

Although the overall CO_2_ conversion trends for the Fe_75_–Ru_25_/Ce–Al_2_O_3_ catalyst (Figure 8a) are consistent across the two independent runs, some differences are evident, particularly at lower temperatures. For example, Run 2 shows higher conversion at 500 °C compared to Run 1, while the profiles converge at higher temperatures (>650 °C). These variations likely stem from experimental factors such as slight differences in catalyst packing, heat transfer, or gas flow dynamics between runs, which can influence the onset of CO_2_ activation during temperature-programmed experiments. Additionally, the RWGS reaction is highly sensitive to surface conditions, and minor changes in catalyst morphology or oxidation state between runs may affect low-temperature activity.^31^

Aging tests of the two best-performing catalysts (Fe_75_–Ru_25_/Ce–Al_2_O_3_ and Fe_75_–Ru_25_/La–Al_2_O_3_) are shown in Figure 8b. It should be noted that these tests were performed sequentially after the thermal cycling tests. Initially, within the first hour, both catalysts exhibited CO_2_ conversions like those observed in cycle 3 of the temperature ramp tests −14% for Fe_75_–Ru_25_/La–Al_2_O_3_ and 16% for Fe_75_–Ru_25_/Ce–Al_2_O_3_. However, the catalytic activity gradually decreased over time (i.e., 60 h), eventually stabilizing near the CO_2_ conversions observed in the first temperature ramp cycle – 5% for Fe_75_–Ru_25_/La–Al_2_O_3_ and 9% for Fe_75_–Ru_25_/Ce–Al_2_O_3_. Contrary to the observed increase in activity over the three cycles, similar factors could also be causing the decrease in activity over time (i.e., changes to particle size, surface morphology, oxidation state and available active sites). Changes in particle size was observed for both catalysts, as shown in the TEM images shown in Figure 2. The Fe_75_–Ru_25_/La–Al_2_O_3_ showed a larger increase in average particle size (22.1 nm) than Fe_75_–Ru_25_/Ce–Al_2_O_3_ (6.4 nm), which likely contributed to its greater decline in activity for Fe_75_–Ru_25_/La–Al_2_O_3_. Additionally, from the TPR profiles postreaction (Figure 3d), it is evident that a significant change occurred for both Fe_75_–Ru_25_/La–Al_2_O_3_ and Fe_75_–Ru_25_/Ce–Al_2_O_3_, showing a loss of RuO_2_ and Fe_2_O_3_/Fe_3_O_4_ active sites.

Overall surface area postreaction (as shown in Table 1) did not significantly change for the Fe_75_–Ru_25_/Ce–Al_2_O_3_ catalyst and only decreased slightly for Fe_75_–Ru_25_/La–Al_2_O_3_, therefore surface area is not the main contributing factor for the decline in activity. Furthermore, for the Fe_75_–Ru_25_/Ce–Al_2_O_3_ catalyst, it is possible that excessive amounts of Ce­(III) (as shown by XPS in Figure 4g) may have formed over the course of the reaction, reducing the support material’s ability to reoxidize, causing a reduction in catalytic activity. Nevertheless, CO selectivity remained close to 100% throughout the aging test, indicating that the RWGS pathway was strongly favored at 500 °C, even as CO_2_ conversion gradually declined over time.

To evaluate whether carbon deposition contributed to the observed catalyst deactivation, thermogravimetric analysis (TGA) was performed on the spent samples following the stability tests. The TGA profiles (provided in the Supporting Information – Figure S2) showed negligible weight loss across the temperature range typically associated with the oxidation of carbonaceous species (i.e., 400 to 700 °C). This indicates that carbon deposition is not a significant factor in the deactivation process.

Further confirmation was obtained from X-ray photoelectron spectroscopy (XPS) analysis. The surface carbon content of the spent catalysts did not exhibit a notable increase compared to the fresh samples, supporting the conclusion that carbon accumulation on the catalyst surface is minimal. Taken together, these findings rule out carbon deposition as a plausible deactivation mechanism.

## 4 Conclusions

This work establishes a new performance window for supported Fe_*x*_-Ru_*y*_ catalysts (2 wt %) catalysts under RWGS conditions, particularly at lower temperatures (∼500 °C), where high CO selectivity is achieved at significantly reduced Ru loadings compared to prior studies that focused on methanation or single-support systems. For all supported Fe_75_–Ru_25_ catalysts, CO selectivity was found to be approximately 100% across the evaluated temperature range (300 to 800 °C). By evaluating Fe–Ru catalysts synthesized on a series of supports spanning different oxygen-vacancy concentrations, we provide insights not previously reported on how oxygen vacancies and metal–support interactions govern catalyst reducibility, stability, and performance. Key findings include:

- Fe:Ru ratio of 75:25 delivers near-equilibrium CO_2_ conversion at 500 °C while maintaining ∼ 100% CO selectivity.
- Ce-doped Al_2_O_3_ emerged as the most effective support For Fe_75_–Ru_25_ (i.e., CO_2_ conversion was 22% at 500 °C), combining high dispersion of active sites with balanced SMSI for superior activity and thermal stability.
- Oxygen vacancies enhance CO_2_ activation and CO desorption, but excessive SMSI (e.g., Sm-CeO_2_) can hinder low-temperature performance.
- Postreaction TPR and TEM revealed structural evolution, with Fe_75_–Ru_25_/Ce–Al_2_O_3_ showing minimal agglomeration compared to Fe_75_–Ru_25_/La–Al_2_O_3_ and Fe_75_–Ru_25_/Sm-CeO_2_.
- Synergistic Fe–Ru interaction improves RWGS kinetics and suppresses methanation, supported by activation energy trends (56–120 kJ/mol).
- Long-term stability tests confirmed Fe_75_–Ru_25_/Ce–Al_2_O_3_ retains higher activity than La–Al_2_O_3_, while TGA and XPS ruled out carbon deposition as a deactivation mechanism.

Collectively, these results advance fundamental understanding of the role of oxygen vacancies and bimetallic synergy in Fe/Ru-based catalysts and provide practical design principles for developing robust, scalable CO_2_ utilization technologies.

## Supplementary Material



## Notes

Additional experimental and characterization data for the Fe/Ru-based catalysts are provided. Figure S1 compares CO_2_ conversion profiles of a representative catalysts under different heating protocols using the experimental system. Figure S2 presents TGA results for Ce–Al_2_O_3_ and La–Al_2_O_3_ supported Fe_75_–Ru_25_ catalysts, highlighting differences in water retention and surface stability before and after aging (PDF)
