# Supplementary material for: Role of Oxygen Vacancies in Fe/Ru-Based Catalysts for the Reverse Water Gas Shift Reaction: Performance and Characterization
Source: ACS Omega. 2026 Jan 27;11(6):10304–19. doi: 10.1021/acsomega.5c11416 (PMC12917709; doi:10.1021/acsomega.5c11416)
Supplement: Supplementary file 1 [file ao5c11416_si_001.pdf]

Role of Oxygen Vacancies in Fe/Ru-Based Catalysts for the Reverse Water Gas Shift Reaction: Performance and Characterization

Holly Dole*, Gianni Caravaggio, Najmeh Ahledel, Ramzi Aoun, Hamid Radfarnia, Kourosh E. Zanganeh

Government of Canada, Natural Resources Canada, 1 Haanel Drive, Ottawa, Ontario, K1A 1M1

*corresponding author, email address: holly.dole@nrcan-rncan.gc.ca

Supplementary Information

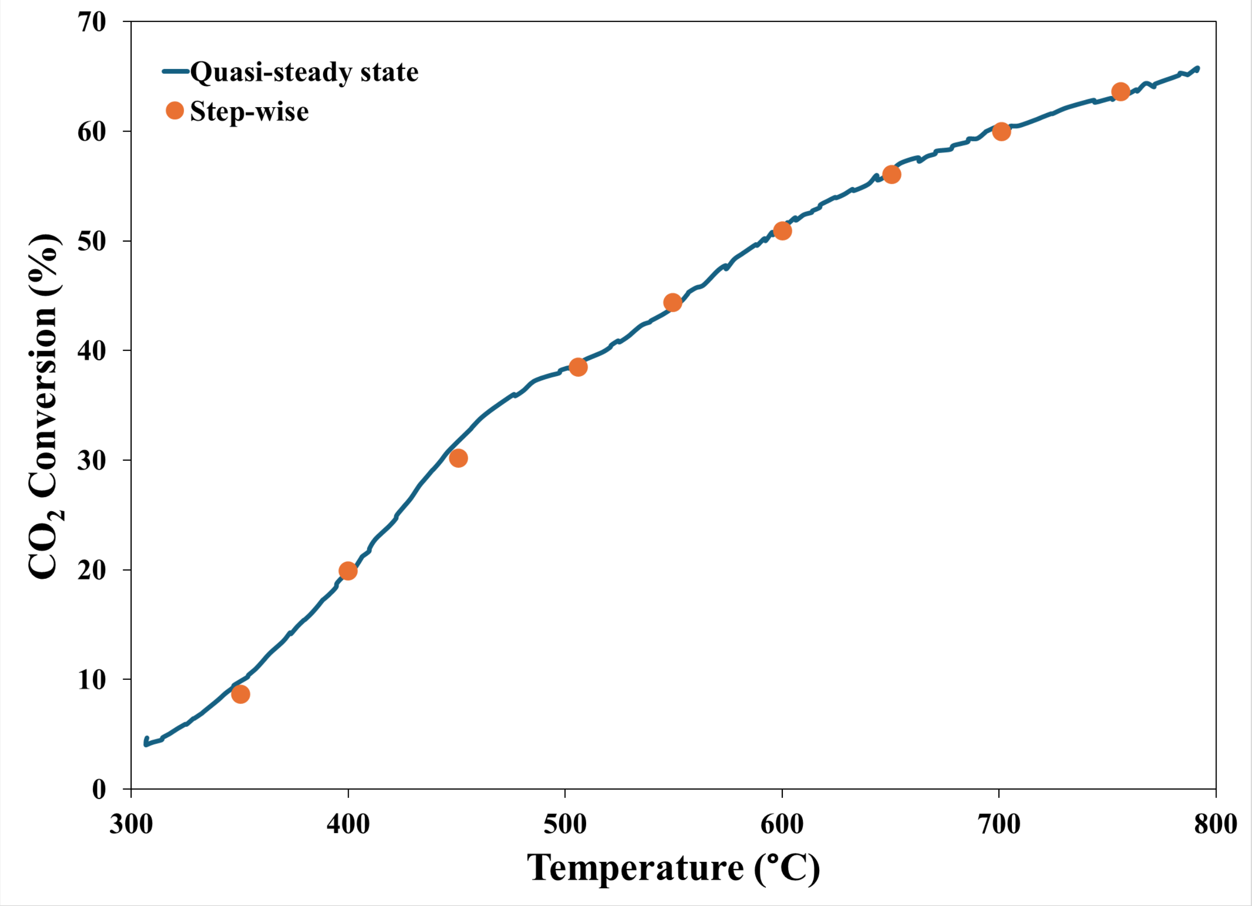

Figure S1:
CO_2 conversion as a function of temperature for representative catalyst ($Ru_{45}Fe_{55}/Sm\text{-}CeO_2$), comparing step-wise temperature increases every 50°C versus ramping at 2°C/min.

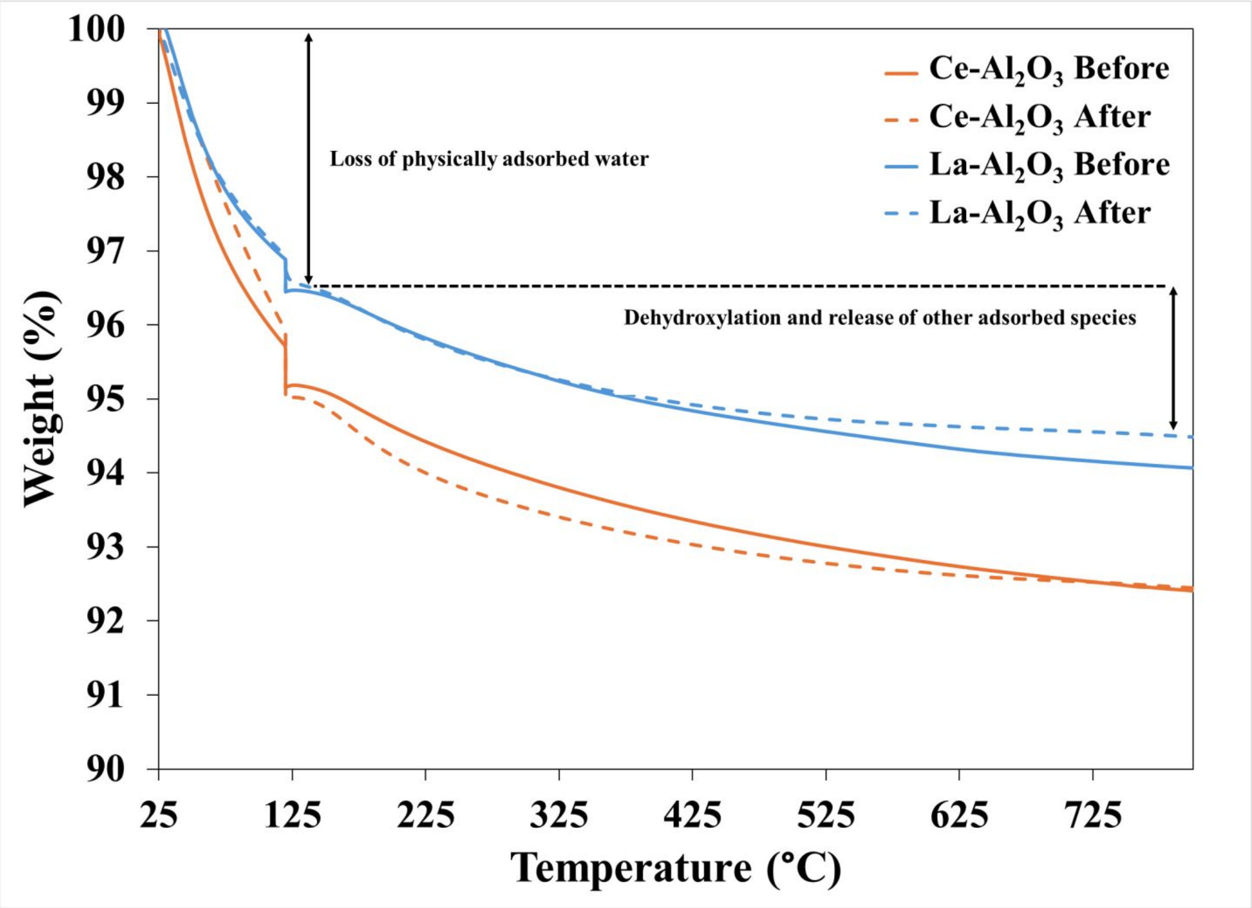

Figure S2:

Thermogravimetric analysis (TGA) of Ce–Al_2O_3 and La–Al_2O_3 catalysts before and after ageing. Both materials exhibit an initial sharp weight loss between 25 °C and ~150 °C, corresponding to the removal of physically adsorbed water and surface hydroxyl groups. A gradual weight decrease occurs at higher temperatures (150–725 °C), attributed to dehydroxylation and the release of residual adsorbed species. Ce–Al_2O_3 shows a greater overall weight loss compared to La–Al_2O_3, indicating higher water retention and more labile surface species. The smaller weight loss observed after ageing suggests reduced water uptake and surface densification following thermal treatment.
